# Quantification of within-sample genetic heterogeneity from SNP-array data

**DOI:** 10.1038/s41598-017-03496-0

**Published:** 2017-06-12

**Authors:** Pierre Martinez, Christopher Kimberley, Nicolai J. BirkBak, Andrea Marquard, Zoltan Szallasi, Trevor A. Graham

**Affiliations:** 1 Université de Lyon, Université Claude Bernard Lyon 1, INSERM 1052, CNRS 5286, Centre Léon Bérard, Cancer Research Center of Lyon, Lyon, France; 2 Evolution and Cancer laboratory, Centre for Tumour Biology, Barts Cancer Institute, Queen Mary University of London, Charterhouse Square, EC1M 6BQ London, UK; 3 Centre for Biological Sequence Analysis, Technical University of Denmark, Lyngby, Denmark; 4 Children’s Hospital Informatics Program at the Harvard–MIT Division of Health Sciences and Technology (CHIP@HST), Harvard Medical School, Boston, MA USA

## Abstract

Intra-tumour genetic heterogeneity (ITH) fosters drug resistance and is a critical hurdle to clinical treatment. ITH can be well-measured using multi-region sampling but this is costly and challenging to implement. There is therefore a need for tools to estimate ITH in individual samples, using standard genomic data such as SNP-arrays, that could be implemented routinely. We designed two novel scores *S* and *R*, respectively based on the Shannon diversity index and Ripley’s L statistic of spatial homogeneity, to quantify ITH in single SNP-array samples. We created *in-silico* and *in-vitro* mixtures of tumour clones, in which diversity was known for benchmarking purposes. We found significant but highly-variable associations of our scores with diversity *in-silico* (p < 0.001) and moderate associations *in–vitro* (p = 0.015 and p = 0.085). Our scores were also correlated to previous ITH estimates from sequencing data but heterogeneity in the fraction of tumour cells present across samples hampered accurate quantification. The prognostic potential of both scores was moderate but significantly predictive of survival in several tumour types (corrected p = 0.03). Our work thus shows how individual SNP-arrays reveal intra-sample clonal diversity with moderate accuracy.

## Introduction

Cancer is a disease in which malignant cells evolve from normal cells within a multicellular organism. Technological advances such as next generation sequencing or single nucleotide polymorphism (SNP) arrays have revealed the (epi)genetic mutations involved in malignant transformation, and highlighted the array of (epi)mutations involved in carcinogenesis^1, 2^. Because tumorigenesis and subsequent cancer development follow an evolutionary process^3^, the continuous evolution of malignant cell populations will inevitably give rise to intra-tumour heterogeneity (ITH)^4^. ITH has been documented in different tumour types using various experimental techniques and designs^5–7^. Its clinical implications are multiple: diversity fosters resistance via (epi)genetic alterations present in subpopulations (or “subclones”), standard single-biopsy sampling will incompletely describe tumours, and biomarkers based on them may therefore lack precision to guide therapeutic decisions^8, 9^.

To date, multiple methods have been developed to reconstruct the clonal structures of tumours^10–12^, while others have focused solely on quantifying the degree of ITH in various data types^13–16^; these methods often rely on obtaining multiple samples from each tumour. Multi-region studies present prohibitive logistic difficulties, both in terms of access to multiple tumour samples from patients and in the increased costs and complexity of data analysis. Moreover, there is a wealth of publicly available genomic data derived from a single sample per tumour^17, 18^. There is therefore a need for methods to accurately measure the heterogeneity of a tumour from a single sample.

Quantification of ITH is a proxy for the evolvability of a tumour. A more diverse tumour is more likely to contain cells that are pre-adapted to a new selective pressure (e.g. chemotherapy). Indeed, recent studies designed different ways to measure ITH and reported it was positively associated with poorer survival. Mengelbier *et al*. used the presence of subclonal alterations from SNP array data in paediatric cancer^19^, while other groups inferred ITH from paired sequencing and SNP-array data in pan-cancer analyses^20, 21^. Mroz *et al*. developed a statistic based on the deviation in mutant allele frequencies from single-sample sequencing data, which was linked to poor outcome in head & neck cancers^22^. Using imaging, and therefore phenotypic rather than genetic data, Yuan *et al*. measured the cellular heterogeneity of tissue sections and integrated it in a prognostic tool for ER- breast cancers^23^. However, there are no standards yet for ITH metrics and it is not clear how resilient the proposed measures are to differences in sample quality and technical noise. Importantly for this study, SNP arrays (and more recently low-pass whole genome sequencing to study copy-number variation^24^) remain a robust, cost-efficient way to obtain genomic data routinely and large cohorts analysed using this technology are publicly available. Yet, no method exists to quantify ITH in single SNP array samples.

Here we derived two methods to quantify ITH from individual SNP-array cancer samples and assessed their accuracy and usefulness. We first used publicly available data to generate synthetic copy number profiles, and used these profiles to generate *in silico* mixtures of related (sub)clones upon which we assess the performance of the ITH measures and also their sensitivity to variations in cellularity. We next created *in vitro* mixtures of clonally derived cell lines to further assess the performance of the ITH scores on real SNP-array data. We finally compared our scores to an existing method before investigating their prognostic potential *in vivo* in over 5,000 clinical samples across 16 cancer types.

## Results

### Novel diversity scores

We designed two scores to estimate genetic diversity from single-sample SNP array data. They are based on the standard logR ratios (hereafter abbreviated to logR) and B allele frequencies (BAF). logR ratios are the log2 of the ratio between the observed copy number (CN) to the expected CN (2 copies in a normal diploid genome). BAF indicate the ratio of an allele arbitrarily defined as ‘B’ allele to the ‘A’ allele for known single nucleotide polymorphisms (SNP). BAF are mirrored the 0.5 axis to mediate the arbitrary A and B allele definition for gain and loss events spanning multiple SNP locations.

#### Shannon diversity-based score: *S*

Segments with identical CN are expected to have the same logR value, and thus the distribution of logR values should contain multiple ‘peaks’ each corresponding to a different CN value. Subclonal CN alterations are expected to create outliers outside these peaks and thus lead to a higher entropy of the logR distribution. To calculate the entropy of the distribution, segmented logR values are grouped into *n* equally sized bins spanning the entire distributions of logR values. The bin size is therefore (max(logR) − min(logR))/*n* and the lowest bin starts at the minimum logR value observed in the sample. The default number of bins was set to 10. Each segment is assigned to a bin and the diversity score is given by the calculation using the *vegan* R package^25^. The Shannon diversity index *S* is calculated from the proportion of segments whose logR value fall into bin *i* denoted by *p*_*i*_ as given by formula (1).

1 $${\rm{S}}=\underset{i}{\overset{n}{-\sum }}pi\times \,\mathrm{ln}(pi)$$

#### Ripley’s L-based score: *R*

As for the S score, segments with the same allele-specific CN are expected to cluster together in a 2-dimensional space whose axes are the logR and BAF values. Subclonal events will create outliers deviating from the clusters corresponding to the clonal BAF/logR values. Ripley’s L score quantifies how randomly a set of points are distributed across a space: subclonal events will create more isolated points in BAF/logR space and thus lower the value. To limit the search space to segments that are very close in space, as expected when segments have the same CN, the default maximum radius of the Ripley’s L statistic was thus set to 0.05. Segmented logR and BAF values from the sample were both linearly normalised to range from 0 to 1, each segment corresponded to a point on a two-dimensional plane of which the normalised logR and BAF were the axes. Ripley’s K-function *K*(*r*) is a measure of deviation from spatial homogeneity (points are randomly distributed across the space) and the L-function *L*(*r*) is its variance stabilised transformation for a given radius *r*. Their formulas are given in (2) and (3).

2 $$K(r)=\frac{\lambda }{n\,\times (n-1)}\sum _{i\ne j}I({d}_{{ij}}\le r)\times {{\rm{e}}}_{{\rm{ij}}}$$

3 $$L(r)=\sqrt{\frac{K(r)}{\pi }}$$

where *λ* is the area of the window, *n* the number of points, *d*_*ij*_ is the euclidean distance between points *i* and *j* and *e*_*ij*_ is the isotropic edge correction weight. The sum is taken over all ordered pairs of points *i* and *j* and *I*(*d*_*ij*_ <  = *r*) is an indicator that equals 1 if *d*_*ij*_ is less than or equal to *r*. We used the *spatstat* R package^26^ to calculate the difference between the reported *L*(*r*) values and the theoretical expected values for all radiuses from 0 to a maximum radius *r*_*max*_ in 0.001 increments. *R*, the sum of all the differences, was taken as the diversity index. Note that spatial homogeneity is expected when points are located at random on the plane, a lower *R* value is therefore expected as genetic diversity increases.

Both measures rely on the use of segmented SNP-array data but do not require estimation of absolute (or allele specific) CNs. They however both suffer from the fact that samples with more extreme CNs will have broader logR distributions and more variable BAFs, regardless of the cellularity of each alteration. Each measure is independently calculated on a per sample basis. To limit the influence of the less reliable shorter segments, only segments of 100 probes or more are taken into account when computing diversity measures. Finally, both scores include normalisation steps for the logR and BAF values, in an attempt to minimise the influence of different levels of cellularity.

### Synthetic copy number profiles and *in-silico* mixtures

**Figure 1:**
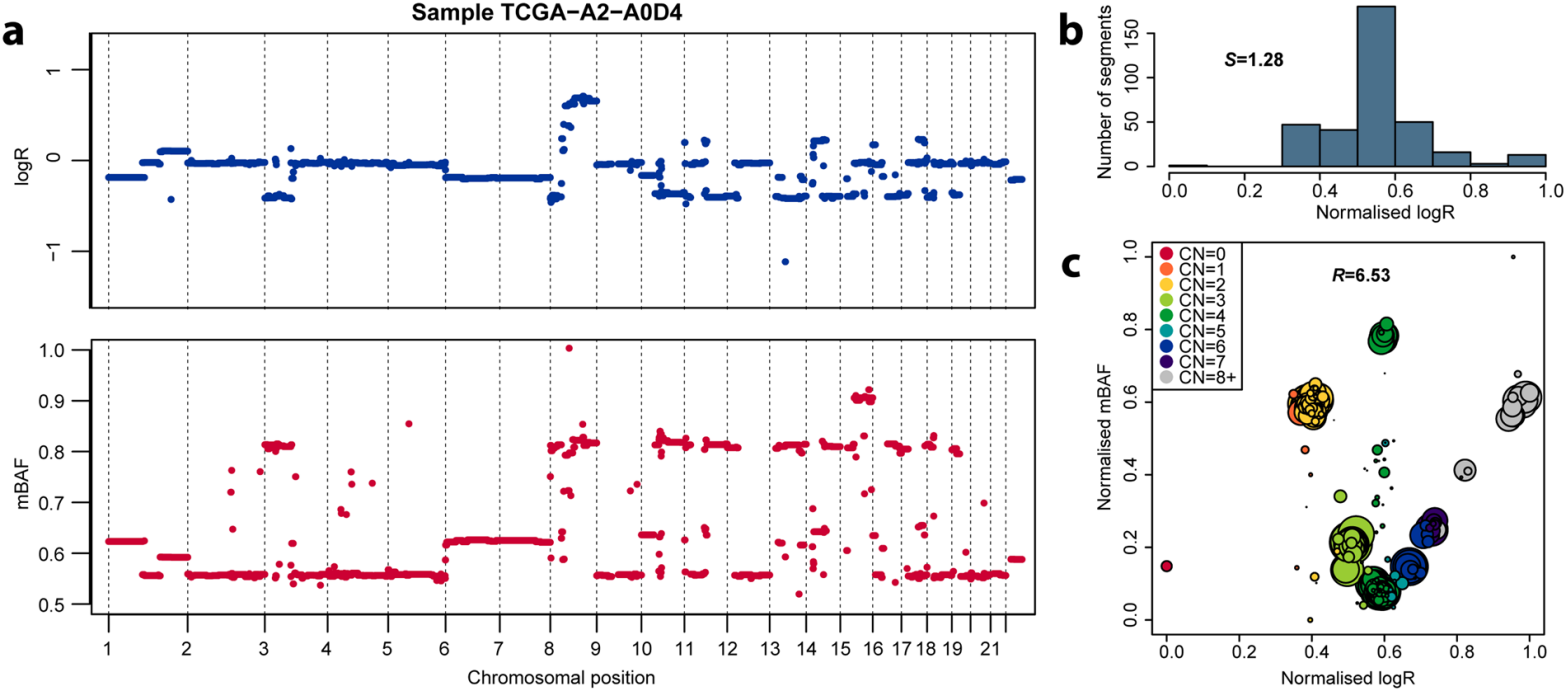
Single-sample SNP array diversity measures. TCGA breast cancer sample TCGA-A2-A0D44 was used as an example, only segments with length >100 probes are analysed. Normalisation was performed so that both logR and mBAF values would range from 0 to 1. (**a**) Segmented logR and mirrored B allele frequency (mBAF) data. (**b**) 10-bin histogram of normalised logR values. (**c**) 2D plot of all segments. Circle are centred on the logR and mBAF values of each segment, their sizes are proportional to segment length. Colours indicate the total copy number of each segment, as reported by ASCAT.

**Figure 2:**
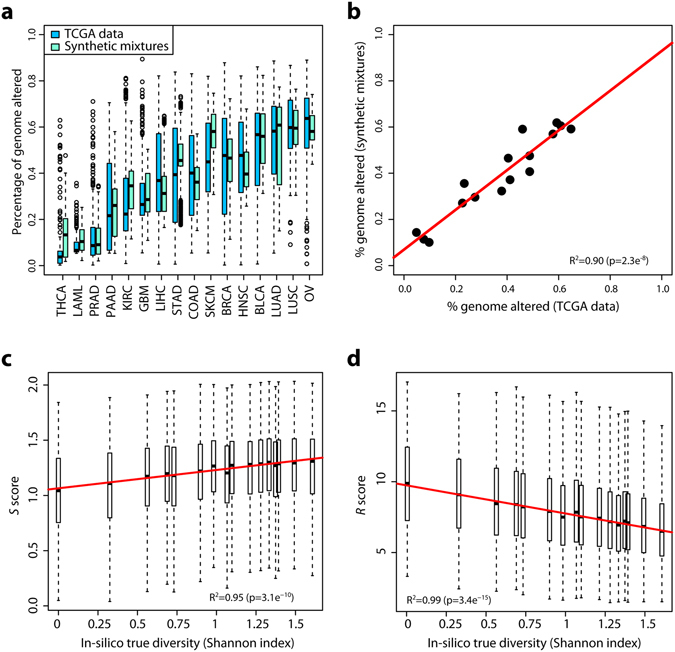
*In-silico* clonal mixtures. (**a**) Distributions of the percentage of altered genome in samples from the TCGA cohorts (blue) and the computationally simulated copy number profiles (cyan). (**b**) Median percentage of genome altered in TCGA and simulated data across cancer types. Each point is a different cancer type and the red line indicates the linear fit between the two datasets. (**c**,**d**) Correlation of the *S* score (**c**) and *R* score (**d**) with the true diversity in *in-silico* mixtures. the red line indicates the linear fit of the median scores at each possible diversity measure. Boxplots: boxes represent the middle quartiles, whiskers indicate the 95% confidence intervals, horizontal bars show the median and outliers are highlighted by circles.

We investigated the relevance of the two proposed genetic diversity scores; 1) a measure based on the Shannon diversity of logR values referred to as the *S* score, and 2) a measure based on the Ripley’s L statistic of spatial homogeneity based on logR and BAF values, referred to as the *R* score (Fig. 1). We first generated in silico datasets from publicly available SNP array data on 16 sets from The Cancer Genome Atlas (TCGA) corresponding to different cancer types. We generated 10 “clonal” CN profiles per set, taking into account the distributions of ploidy and percentage of altered genome in each set. An additional 4 “subclonal” profiles were derived from each clonal profile based on an expected divergence of 15% ± 5% of the genome (see Methods). The median percentage of genome altered of the synthetic mixtures and TCGA data were correlated on a per-cancer type basis (R^2^ = 0.90, p < 0.001, Fig. 2a,b), indicating that we had generated set of synthetic profiles resembling publicly available cancer data. A total of 707,200 mixtures were generated *in-silico* using different combinations of clonal and subclonal profiles at 4 different levels of cellularity (percentage of tumour cells in a sample): 20%, 40%, 60% and 80%. The Shannon diversity index of the clonal composition of each mixture was utilised to quantify the heterogeneity of each mixture (“true diversity” hereafter).

### Correlation between diversity scores and expected heterogeneity in *in-silico* clonal mixtures

We compared the true diversity of *in-silico* mixtures (quantified using the Shannon diversity index), to the *S* and *R* diversity scores calculated on each mixture. At high cellularity (80%), the median *S* and *R* were respectively highly correlated and anti-correlated to the median Shannon diversity index of each mixture respectively (R^2^ = 0.95 and R^2^ = 0.99; both p < 0.001; Fig. 2c,d). However, the variability at each expected level of *in-vitro* diversity was high, meaning confident identification high diversity samples from low diversity ones was challenging despite the correlation. Indeed, the fit of all points rather than of the medians were poor although the (anti-)correlation was highly significant (R^2^ = 0.01, p < 2e^−16^ in both cases). Notably the *R* score displayed a more pronounced slope with lesser variability than the *S* score.

Within the *in-silico* mixtures, the percentage of genome altered (see Methods) was highly correlated to the *S* score and anti-correlated to the *R* score (p < 0.001, Supplementary Figure 2). However, the median percentage of genome altered was not correlated to the true Shannon diversity of the *in-silico* mixtures (R^2^ = 0.21, p = 0.07, Supplementary Figure 3). Our synthetic dataset analysis therefore suggests that the overall level of genetic alterations present in the genome is an inadequate way of assessing the clonal diversity of a tumour from a single SNP array sample.

### Influence of heterogeneity in sample cellularity in *in-silico* clonal mixtures

We next considered the effect of cellularity on our diversity measures. When looking at low cellularity mixtures (cellularity, or cancer cell fraction = 0.2), the median S and R scores were still significantly correlated with the true diversity (R^2^ = 0.87 and R^2^ = 0.92 respectively; both p < 0.001; Supplementary Figure 4). The fit and slopes were lower than in high cellularity samples in both cases, indicating that quantifying diversity in lower cellularity samples is more challenging.

**Figure 3:**
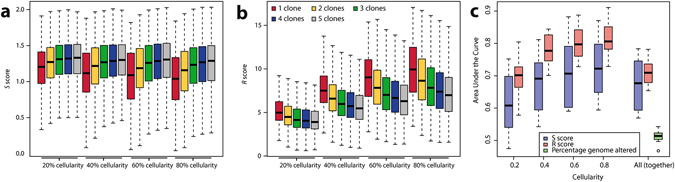
Accuracy of the *S* score in polyclonality detection *in-silico*. (**a**,**b**) Distribution of *S* and *R* scores per different levels of cellularity (tumour cell fraction in a sample), according to the number of clones present in *in-silico* clonal mixtures. (**c**) Distribution of the Area Under the Curve in all 16 sets for both *S* and *R* scores at different cellularity levels. “All” indicates that samples with 0.2, 0.4, 0.6 and 0.8 cellularity were all included in the dataset. The percentage of genome altered was calculated on.

Furthermore, varying levels of cellularity are expected within real clinical cohorts, which could prove problematic if the scores are highly dependent on sample cellularity. Indeed, we observed that the R score was strongly affected by different levels of sample cellularity (Fig. 3a,b). We used Area Under the Curve (AUC) analysis on a per-set basis to assess the power to predict whether a mixture of any cellularity was monoclonal (1 clone only) or polyclonal (2 clones or more). When analysing samples of identical cellularity, the R score was a powerful predictor with the median AUC ranging between 0.70 and 0.81, while the S score AUC ranged from 0.61 to 0.72 (Fig. 3c). However, when samples with different cellularity levels were included, the performance of both scores were comparable (p = 0.85, paired t-test), with the S and R scores respectively achieving a median AUC of 0.68 and 0.67. These data indicate the confounding influence of cellularity on ITH quantification. The percentage of genome altered, was not found to bear any predictive value in our synthetic data (median AUC of 0.51). We however note that this dataset assumed perfect detection of CNAs rounded to the closest integer, while this could in practice affect genotype prediction.

### *In-vitro* cellular evolution and isogenic clonal mixtures

**Figure 4:**
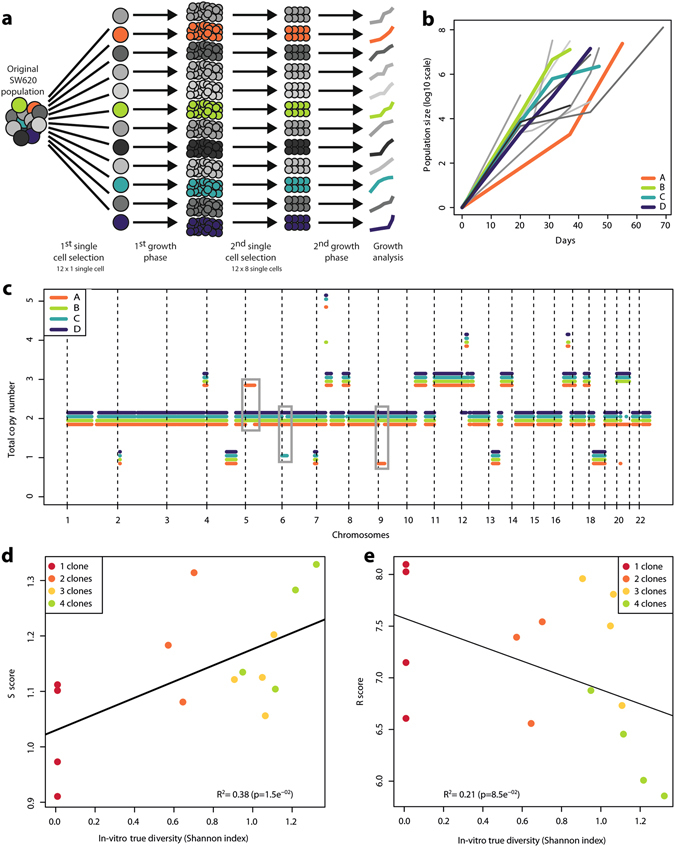
*In-vitro* clonal mixtures. (**a**) Experimental procedure. (**b**) Growth curves of 12 single cell cloned isogenic cell lines. In colour, the 4 cell lines that were selected for further characterisation and *in-vitro* mixing of DNA. (**c**) Total copy number profiles of A, B, C and D, the 4 selected cell lines. Grey rectangles highlight regions where one profile showed marked divergence with the others. Vertical dashed lines indicate chromosome boundaries. (**d**,**e**) Correlation of the *S* score (**c**) and *R* score (**d**) with the true diversity in *in-vitro* mixtures. Black lines indicate the linear fit between the scores and the expected diversity.

Two rounds of single cell cloning were used to generate twelve isogenic cell lines from the chromosomally unstable SW620 colorectal cancer cell line (Fig. 4a, see Methods). We analysed the proliferation rate of the 12 isogenic cell populations, and found they exhibited different growth patterns. We selected 4 clones (designated A, B, C and D), because of their apparent different growth dynamics (Fig. 4b). DNA was extracted from each clone, and we created 12 mixtures consisting of DNA from 2 to 4 of these cell populations at different proportions. We calculated the expected diversity of each mixture (“true diversity”), using the Shannon diversity index based on the frequency of each population (Supplementary Table 2). We defined the genomic profiles of the 12 mixtures and of the 4 initial cell populations by SNP array analysis. Mixture 8 failed quality control (call rate < 0.95; all other call rates > 0.98) and was excluded from subsequent analyses. Although the CN profiles of the initial cell lines showed little divergence, some large CN alterations (CNAs) were specific to a unique cell line: 5q gain and 9q loss in A; 6q loss in C (Fig. 4c). In total, 7.5% of the genome (201 out of 2,755 Mb) analysed presented a CN state that was not uniform across all cell lines.

### Correlation with genetic diversity in *in-vitro* clonal mixtures

We calculated the *S* and *R* scores on the *in-vitro* mixtures and compared the outcome with the true Shannon diversity. Both scores were correlated with the true diversity, although it was statistically significant only for the *S* score and borderline for the *R* score (p = 0.015 and p = 0.085, respectively, Fig. 4d,e), and the R^2^ values (percentage of variance explained) were low (0.38 and 0.21, respectively). To assess whether the low divergence observed between the 4 unmixed profiles influenced the results, we performed a second analysis restricted to the 7.5% of the genome that was divergent. We however report similar results in this design, this time with significance being weak for S and moderate for R (p = 0.16 and p = 0.04, respectively; Supplementary Figure 5).

Despite the limited inter-sample variability, these results confirmed that both scores were informative on the clonal heterogeneity of ‘real’ single samples analysed by SNP array. In addition, we calculated the percentage of genome altered in all samples, which was not significantly related to the expected diversity (R^2^ = 0.06, p = 0.388, Supplementary Figure 6). The AUCs to distinguish polyclonailty from monoclonality obtained *in-vitro* were 0.81 and 0.77 respectively for *S* and *R*, indicating reasonable power to discriminate between monoclonal and polyclonal samples *in-vitro* (Supplementary Figure 7). The percentage of genome altered achieved a lower AUC of 0.62.

### Calibration of diversity scores to *in-silico* and *in-vitro* data

Both scores rely on a tunable parameter: the number of bins for the S score and the maximum radius size for the R score. We therefore used our *in-silico* and *in-vitro* data to define the most appropriate parameters for use on real data. We selected 10 possible values for the number of bins (6 to 24) in the S score, and 7 for the maximum radius (0.025 to 0.25) in the R score, then analysed the correlation between the obtained scores and the true diversity in the *in-silico* and *in-vitro* datasets (Supplementary Figures 8 and 9). We furthermore analysed their power to discriminate between monoclonal and polyclonal samples using AUC calculations. The synthetic data suggested that increasing the number of bins by increments of 2 from 6 to 24 bins would yield S scores more correlated and more predictive with each increment. The *in-vitro* data however peaked sharply at 14 bins before losing power (likely due to the small number of divergent CNAs amongst these samples). For the R score, increasing the maximum radius size led to gradually worse anti-correlation and predictive power in the *in-silico* data, while the *in-vitro* data registered poor performance for the shortest radius (0.025). We therefore empirically decided to use 12 bins for the S calculation, and a maximum radius of 0.05 for the R calculation as optimal parameters for use in real cancer data.

### Association with survival in available clinical datasets

**Figure 5:**
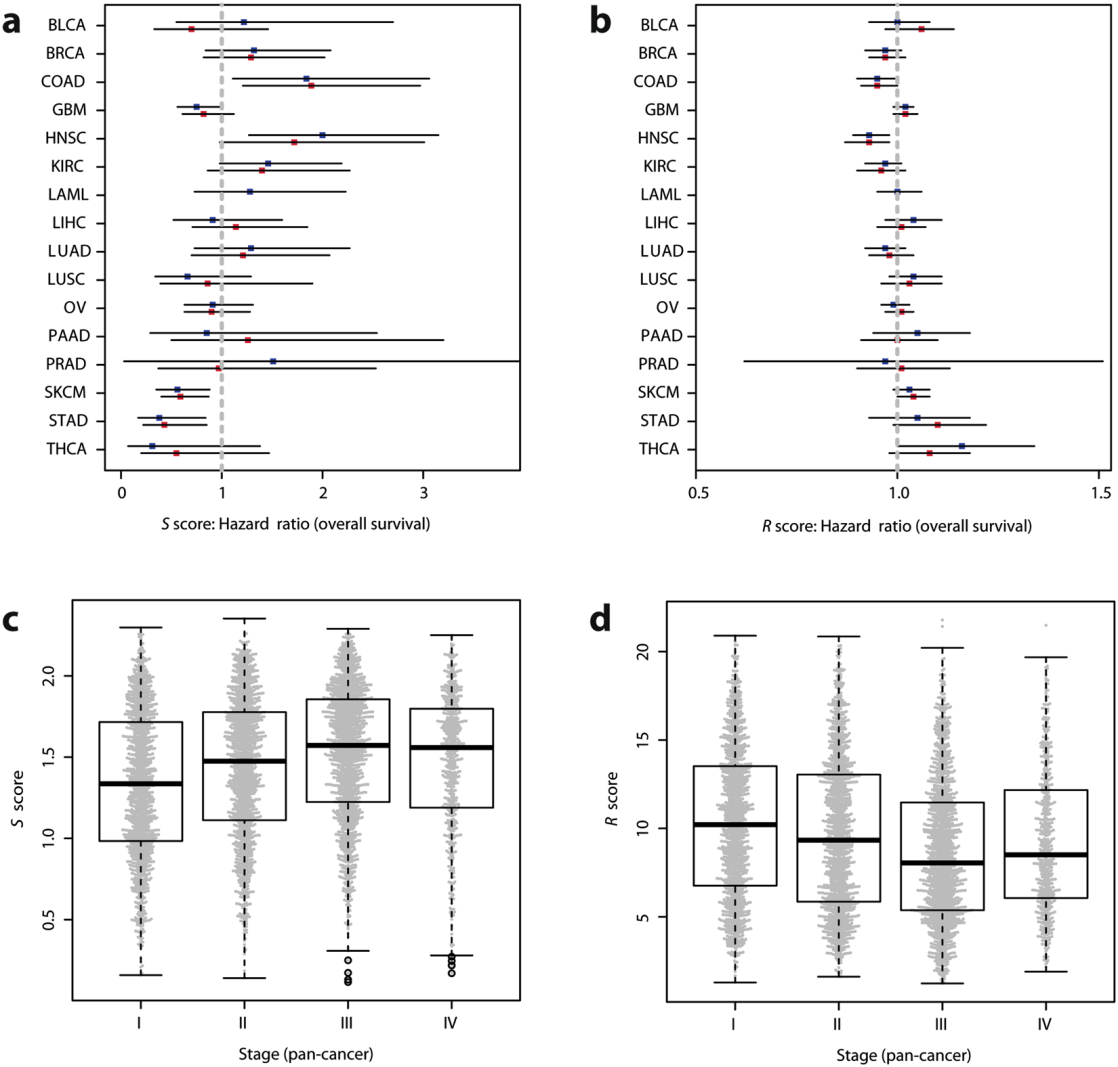
Relationships of the *S* and *R* scores with survival and stage in real cancer data. (**a**,**b**) Hazard ratios and confidence intervals for overall survival Cox proportional based on the *S* score (**a**) and *R* score (**b**) for all cancer types. Blue squares correspond to overall survival and red squares to relapse-free survival. Squares indicate the predicted hazard ratios, horizontal black lines indicate the confidence intervals and the vertical dashed grey lines indicate a hazard ratio of 1. X-axis scales differ. (**c**,**d**) Distributions of the *S* score (**c**) and *R* score (**d**) per cancer stage on a pan-cancer basis (4,363 samples with stage and survival information). Gray dots represent each individual sample. Both p < 0.001 (independence test).

We calculated the *S* score on 5,078 samples with survival data from the TCGA dataset, and investigated its relationship with clinical outcome. We used univariate Cox proportional hazard models to test the relationship in both overall and relapse-free survival data (Supplementary Tables 3 and 4, Fig. 5a,b). In univariate Cox regression, *S* was significantly associated with survival in multiple cancers, though the only association that held to multiple testing was the one between higher diversity and poorer survival (OS and RFS) in head and neck cancers, for both scores (p < 0.05). Furthermore, repeating the analysis including either *S* or *R* and clinical stage (as a categorical variable) in multivariate models indicated that both measures were still significant covariates after correction for stage in head & neck cancers, but not in other tumour types. However, *S* and R were respectively correlated and anti-correlated with stage across tumours (p < 2e^−16^, independence test; Fig. 5c,d, Supplementary Figures 10 and 11), particularly in bladder, breast, colon, head & neck and kidney clear cell cancers. We further investigated a pan-cancer meta-dataset by pooling all samples together and found that *S* was significantly associated with poor relapse-free survival in this dataset (p = 0.001) but not with overall survival, while *R* showed no association.

Both scores were also highly correlated with the percentage of genome altered in all tumours (R² = 0.48, p < 2e^−16^; Supplementary Figure 12), and a survival analysis using the percentage of genome altered yielded comparable results, with significance in multiple cancer types (Supplementary Table 5). We could furthermore verify precedent findings that extreme values for the percentage of genome altered (<25% or >75%) were associated to better prognosis on a pan-cancer basis^20^ (OS p = 2.6e^−6^, RFS: p = 9.2e^−8^; Supplementary Figure 13). This suggests that although *S* and *R* appear to better represent the underlying sample diversity than the percentage of genome altered in *in-silico* and *in-vitro* SNP array data, they do not offer increased prognostic value in clinical samples.

### Comparison to EXPANDS’ number of clones

**Figure 6:**
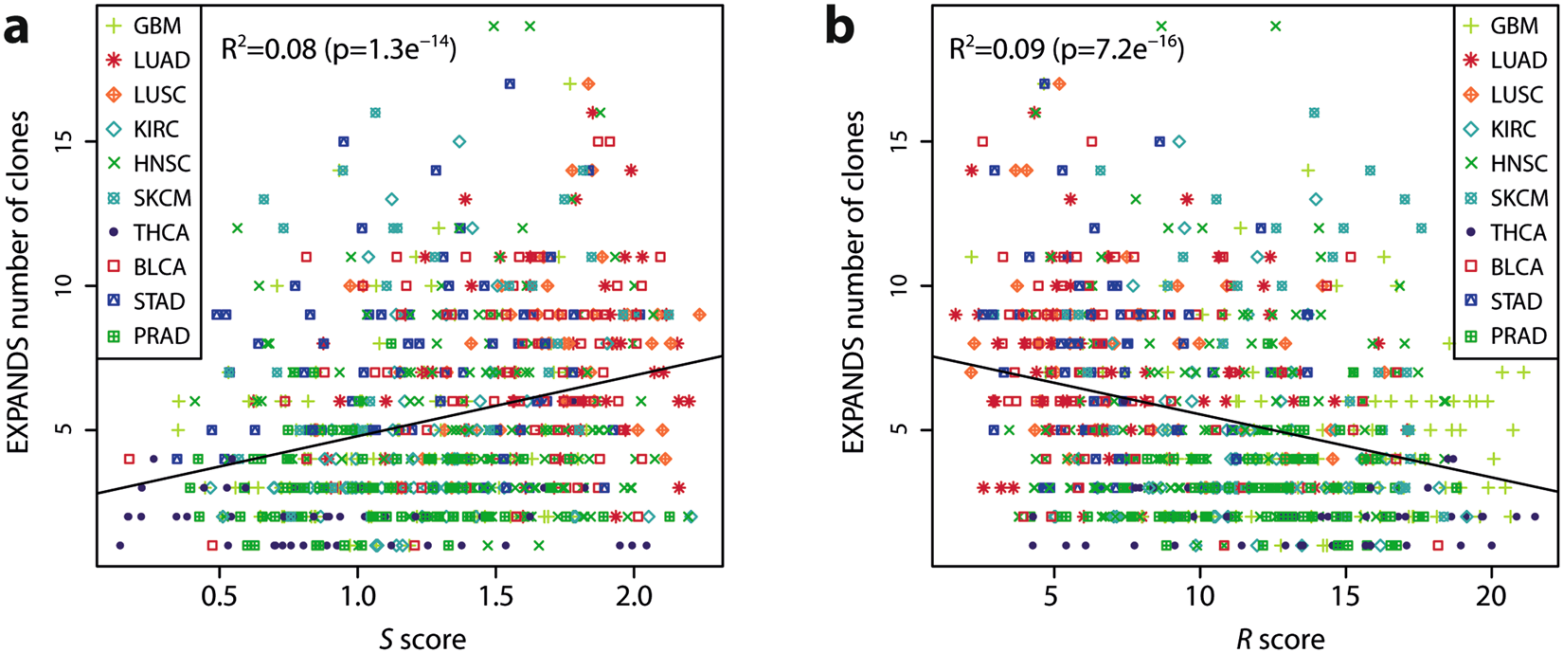
Comparison with other methods and datasets. (**a**,**b**) Correlation between *S* score (**a**), *R* score (**b**) and the number of clones predicted by the EXPANDS software. Different point styles and colours correspond to different cancer types.

To our knowledge, no other method or algorithm has been designed to estimate diversity using only individual SNP array data. However, the EXPANDS software uses single nucleotide allele frequencies from sequencing data in combination with SNP array data to predict the number of clones in a sample^27^. Using TCGA data from 9 tumour types samples where EXPANDS estimated more than 2 clones were present had poorer survival than samples with 2 or fewer large clones^20^. We compared our scores to the EXPANDS number of clones and found that the S and R scores were significantly correlated and anti-correlated, respectively (p < 0.001, Fig. 6) but R^2^ values were low (0.08 and 0.09), indicating high variability. The extra information added by mutation calls from sequencing data, which can more accurately measure clonal frequencies, could however explain the low R^2^ values. This suggests that *S* and *R*, defined on CN alterations, correlate with ITH scores defined on corrected mutational frequencies.

## Discussion

Intra-tumour genetic diversity is a major hurdle to cancer prognostication and therapy and there is no ‘gold standard’ for its quantification, particularly from routine clinical samples. Although the use of multiple samples provides important insight into the clonal architecture of tumours^28^, in practice multiple samples are rarely collected routinely apply because of the inherent difficulties associated with sampling more of the cancer and the prohibitive increased costs of multi-sample assays. SNP-arrays have been widely used in cancer genetic studies to unravel the genomic landscape of multiple tumour types, however typically only one SNP-array is analysed per tumour.

Here we developed two novel metrics to quantify genetic diversity in SNP-array data from single samples and evaluated their performance using *in-silico* and *in-vitro* data, and assessed their prognostic value on publicly available data. We found that our two diversity scores reflected the true diversity of synthetic mixtures. We however highlight the influence of cellularity on both scores, meaning that heterogeneous levels of tumour cell content in cohorts will hamper accurate ITH quantification. The *in-silico* results nevertheless suggested that the R score would be more informative than the S score in cohorts of relatively uniform cellularity, particularly if the latter is high. Finally, we found that the prognostic potential was limited: head and neck squamous cancers were the only cancer type in which a significant association with poor survival was found. This observation nonetheless ties up with previous findings of a sequencing-based ITH measure being correlated to poor survival in this tumour type^22^, highlighting a common behaviour for sequence-level and chromosomal heterogeneity. It suggests that ITH may be particularly relevant for prognosis in head and neck squamous cancers and that further development in routine quantification could be clinically useful in this disease.

While methods exist to infer the clonality of mutations in a tumour^10, 11, 27^, these are generally based on multiple samples. The diversity scores developed in this manuscript differ because they are designed to calculate diversity from a single SNP array sample. Nevertheless, there are inherent limits to a single-sample approach: subclonal events could be invisible without multi-region sampling^29^, meaning that although ITH is present in a tumour, a single biopsy could ‘miss’ the heterogeneous lesion and present a homogeneous profile. Furthermore, SNP arrays are less sensitive to the detection of small clones than sequencing data, particularly when sequencing is performed at high read depth^10, 27^. This sensitivity issues potentially explains why EXPANDS software, that integrates CN and sequencing data, measures much more variability in clonal composition than the SNP-array only methods we present here. In addition, copy number alterations have recently been linked to clonal expansions during punctuated cancer evolution^30, 31^, which could imply they would be less frequently subclonal in small tumour regions than sequence levels variations that accrue continuously^32^.

Finally, the ability to detect subclonal alterations in our method was inevitably correlated to the total number of alterations in a sample – subclones cannot be detected unless they bear unique CNAs. This is a confounding issue, and future improvements of copy-number-based ITH quantification should thus aim at reliably teasing apart low diversity and high CNA load samples from those with high diversity and low CNA load – though this may prove intractable. Consequently, while a single-sample SNP-array diversity score may accurately reflect the diversity of copy-number altered clones present in a sample, it may not reflect the diversity of the tumour as a whole, nor all the biologically relevant genetic diversity in that sample.

Quantification of ITH has direct relevance for both prognostication and treatment choice, but ITH measurement is subject to a compromise between the practicality of clinical implementation and the accuracy and scope of the measurement. Here we have shown that ITH can be quantified from single tumour samples assayed with SNP-arrays, using scores whose computation we have made publically available. Although the scores covaried linearily with clonal diversity in-silico and *in-vitro*, the proposed method may lack precision to reliably tease apart highly altered but stable genomes from highly unstable ones. Despite a very significant association between higher heterogeneity and poor outcome in head and neck cancers, our analysis suggests that the prognostic value of such measurements is generally limited.

## Methods

### Publicly available SNP array data

The raw data from 5,416 tumours in 16 different cancer types were downloaded from The Cancer Genome Atlas (TCGA) between 02/04/2013 and 07/07/2014. Allele-specific copy numbers (CN) were produced using ASCAT^33^ after prior normalisation using the Aroma software^34^, all in the R statistical computing framework^35^. Only segments of length >10 probes were considered. Cancer types and the samples in them are described in Table 1. For cancer type, the distributions of baseline CN and percentage of genome altered was calculated for further use. LogR and BAF data could not be retrieved for 8 out of 5,416 samples.

**Table 1:** TCGA samples.

Set	Type	Number of samples	With OS data	With RFS data	Stage I	Stage II	Stage III	Stage IV	Stage NA
BLCA	Bladder Urothelial Carcinoma	142	141	104	1	44	46	47	4
BRCA	Breast Invasive Carcinoma	935	865	594	156	530	216	15	18
COAD	Colon Adenocarcinoma	396	396	342	66	150	114	58	8
GBM	Glioblastoma Multiforme	454	454	293	0	0	0	0	454
HNSC	Head and Neck Squamous Cell Carcinoma	336	336	207	20	54	54	154	54
KIRC	Kidney Renal Clear Cell Carcinoma	468	467	129	224	54	118	72	0
LAML	Acute myeloid leukemia	154	144	0	0	0	0	0	154
LIHC	Liver Hepatocellular Carcinoma	196	149	136	71	44	56	5	20
LUAD	Lung Adenocarcinoma	367	354	273	198	84	66	19	0
LUSC	Lung Squamous Cell Carcinoma	251	247	147	120	65	58	5	3
OV	Ovarian Serous Cystadenocarcinoma	514	510	290	16	22	398	74	4
PAAD	Pancreatic Adenocarcinoma	63	63	62	5	54	1	3	0
PRAD	Prostate Adenocarcinoma	285	284	234	0	0	0	0	285
SKCM	Skin Cutaneous Melanoma	246	241	235	44	65	92	10	35
STAD	Stomach Adenocarcinoma	170	138	136	31	55	50	22	12
THCA	Thyroid Carcinoma	439	297	237	249	46	94	48	2

Description of the 16 datasets downloaded from the TCGA and the related samples.

### Baseline copy number and percentage of genome altered

The percentage of genome altered was calculated for each sample using segmented allele-specific CN. The baseline copy number state of each sample was defined as the modal CN of the genome. The percentage of genome altered was then defined as the proportion (in base pairs) of the genome that did not match the baseline CN state. In *in-silico* mixtures, real numbers for the average allele-specific CN of each segment weighted by the frequency of each clone were rounded to the closest integer CN, to reflect the standard ASCAT output. These integer CNs were then used for the calculation of the percentage of genome altered.

### Synthetic copy number profiles

Random copy number (CN) profiles were generated for each cancer type in the TCGA data (hereafter referred to as a ‘set’), starting by generating allele-specific CN profiles for 10 “clones” per set (Supplementary Figure 1). Each clone profile *i* was defined by first generating a random baseline allele-specific CN *B*_*i*_ from the set-specific distribution. This takes the form of a pair of integers, defining the major and minor CN most commonly found in the sample’s genome (1 and 1 for a normal genome). Then, the percentage of genome altered *P*_*i*_ (i.e. the percentage of the genome deviating from *B*_*i*_) was randomly generated for each generated profile from a normal distribution, whose mean and standard deviation corresponded to those of the set-specific distribution of the percentage of genome altered. The minimum acceptable value for *P*_*i*_ was set to 2%, so as to ensure a minimal level of abnormality in the profile.

The next step was to define the segmentation of each profile *i* into non-overlapping segments across the genome. This was performed by selecting the segment boundaries of each chromosome (1 to 22) individually from 22 distinct TCGA samples drawn from the same cancer type at random with replacement, then merging all segments into a set *S*_*i*_, in respective chromosomal order, and setting the allele-specific CN of all segments to *B*_*i*_. A random subset of segments *R*_*i*_ was then defined by iteratively sampling segments from *S*_*i*_ (without replacement), until the cumulative length of the segments in *R*_*i*_ was superior to 95% of *P*_*i*_. Finally, each segment *s* of *R*_*i*_ was assigned a CN state, with values taken from a distribution of all CN states from TCGA segments from the same cancer type, whose CN state differed from *B*_*i*_ and whose length was equal to the length of *s* ± 25%.

To model the subclonal architecture of tumours, 4 subclonal profiles were subsequently computed for profile *i*, similar to a progenitor clone and 4 distinct clonally derived subclones that could be present in a tumour. For each subclonal profile *j*, the divergence from the previously obtained ancestor *i* was drawn from a normal distribution centred on 15% ± 5%, with a 1% minimum threshold to ensure the presence of a minimum level of divergence. A set of segments *D*_*i*_ whose CN state in *j* was divergent from *i* was defined and their CN states were assigned using the same method as for *R*_*i*_.

### In-silico mixtures

Clonal combinations of 1 to 5 profiles were selected: all 5 single clones, all 20 combinations of two clones, all 60 combinations of 3 clones, 100 non-redundant randomly selected combinations of 4 and 5 clones, resulting in a total of 285 combinations. For each combination, we defined different assorted frequencies at which to include all clones in the mixtures, depending on the number of clones and always summing to 100%: 3 frequency assortments were chosen for mixtures of 2 clones, 4 assortments were chosen for 3, 4 or 5 clones (Supplementary Table 1). This amounted to a total of 1,105 *in-silico* mixtures for each of the 160 groups of clonally related profiles (1 clonal, 4 subclonal profiles per group). Clonal combinations were therefore order-sensitive, as mixing clones 1, 2 and 3 at respective frequencies 45%, 30% and 25% would yield a mixture different to combining clones 3, 1 and 2 at the same frequencies.

For every combination of *n* clonally related profiles at given frequencies *f*_*n*_, the number of copies *N* of each allele *l* in a segment were calculated as floating point number:

4 $${N}_{l}=\,\sum _{i}^{n}{N}_{{li}}\times {f}_{i}$$

where *i* is the clone identifier from the *n* profiles in the mixtures, *N*_*lsi*_ is the number of copies of allele *l* in segment *s* in profile *i* and *f*_*i*_ is the frequency of profile *i* in the mixture.

The logR *L*(*s*) and mirrored B allele frequencies (BAF) *mB*(*s*) values of each segment *s* were then calculated as follows, according to 4 possible values of cellularity *C*, defined as the fraction of tumour cells in the sample (20%, 40%, 60% or 80%):

5 $$L(s)={\rm{l}}{\rm{o}}{\rm{g}}\,2((({{N}}_{{\rm{a}}}+{{N}}_{{\rm{b}}})\times C+2\times (1-C))/2)$$

where *N*_*a*_ is the real CN (as opposed to nearest integer) of allele A and *N*_*b*_ the one of allele B.

6 $$mB(s)=0.5\,+\Vert 0.5-\frac{({N}_{b}\ast C+(1-C))\,}{(({N}_{b}+{N}_{a})\ast {\rm{C}}+2\ast (1-{\rm{C}}))}\Vert \,$$

The ratio uncorrected for cellularity *N*_*b*_/(*N*_*a*_ + *N*_*b*_) was set to 0.5 when *N*_*a*_ and *N*_*b*_ were equal to 0 (bi-allelic loss of a whole segment), for feasibility. Finally, the final values were taken from normal distributions centred on *L*(*s*) and *mB*(*s*) with standard deviation 0.02, to account for experimental and technical noises.

### Cell culture

To derive clonally related cells with distinct CN profiles, we performed two rounds of single-cell cloning on human colon cancer SW620 cells. Cells were maintained in Dulbecco’s modified Eagle medium (DMEM, D6429, Sigma) supplemented with 10% Foetal Bovine Serum (10500-064, Life Technologies) and 50 U/ml penicillin/streptomycin (15070-064, Life Technologies). The initial population of cells was cultured until 75% confluency.

For the first round of single cell cloning: single cells were isolated using the FACSAria II with the orifice at 100 μm (Beckton Dickinson). Each single cell derived clones were grown until ~75% confluent in 96 well plates, before 12 individual colonies were transferred to individual wells of a 12 well plate and cultured until ~75% confluent, and then each were transferred to an individual 75 cm^2^ flask and again grown until ~75% confluent.

For the second round of single cell cloning: each of the grown-up 12 clones from the previous round was incubated in fresh DMEM with 10 nM Draq5 (62251, Thermo Fisher) at 37 °C for 10 minutes, washed in PBS and individually FACs sorted as previously. From each clone, we isolated individual cells with >2 N nuclear content as determined by Draq5 staining in order to increase the likelihood of selecting cells with increased ploidy. The re-sorted individual cells (8 cells from each of the 12 first-round clones) were then grown until 75% confluent in a 96 well plate. Of those that successfully expanded, one clone from each of the first-round 12 colonies were transferred to individual wells of a 12 well plate and grown until ~75% confluent and then transferred to a 75 cm^2^ flask and again grown until ~75% confluent. DNA was extracted using a DNeasy Blood and Tissue Kit (69505, Qiagen) and quantified on the Qubit. 2.0 Fluorometer (Q32866, Life Techniologies).

### *In-vitro* mixtures and SNP array data

Four of the *in-vitro* clones selected for further study were labelled A, B, C and D. DNA from the 4 clones was combined in different proportions to create 12 distinct mixtures corresponding to different numbers of clones at different concentrations (Supplementary Table 2). The quantity of DNA to be taken from each clone in each mixture was calculated as its frequency in the mixture multiplied by the desired quantity of input DNA (200ng) and the 12 *in-vitro* mixtures were created by pooling the desired clones at the desired concentrations. The 12 *in-vitro* mixtures and an individual sample for each of the 4 clones were then loaded as individual samples on a HumanOmni2.5–8 v1.2 BeadChip (Illumina) run according to the manufacturer’s instructions. LogR values and B allele frequencies (BAF) were extracted from the Illumina GenomeStudio software. One sample (Mixture 8 with all four clones at an equal ratio of 25%) did not pass quality control (call rate <0.98) and was discarded. LogR values were further normalised using the genomic wave correction tool from the pennCNV software suite^36^ and the CN profiles of the 15 remaining samples were computed using ASCAT. The Shannon diversity indices were calculated using the clonal frequencies of each mixture to represent their expected diversity.

### Bioinformatics

All analyses were performed in R. The *pROC* package^37^ was used for ROC analyses. The coin package was used for independence tests^38^.

## Electronic supplementary material

Supplementary Data
