# Supplementary material for: Quantification of within-sample genetic heterogeneity from SNP-array data
Source: Sci Rep. 2017 Jun 12;7:3248. doi: 10.1038/s41598-017-03496-0 (PMC5468233; doi:10.1038/s41598-017-03496-0)
Supplement: Supplementary file 1 — Supplementary Data [file 41598_2017_3496_MOESM1_ESM.pdf]

Quantification of within-sample genetic heterogeneity from SNP-array data

Supplementary Data

Pierre Martinez, Christopher Kimberley, Nicolai J. BirkBak, Andrea Marquard, Zoltan Szallasi, Trevor A. Graham.

Supplementary tables

Supplementary Table 1: Clonal frequencies in in-silico mixtures. Clones are labelled from I to V and order matters (mixing clonally rated profiles 1, 2 and 3 at respective frequencies 45%, 30 and 25% will therefore be different to mixing clones 3, 1 and 2).

Number of clones	Clone I frequency	Clone II frequency	Clone III frequency	Clone IV frequency	Clone V frequency	Shannon Index
1	1	0	0	0	0	0
2	0.9	0.1	0	0	0	0.33
2	0.75	0.25	0	0	0	0.56
2	0.45	0.55	0	0	0	0.69
3	0.75	0.15	0.1	0	0	0.73
3	0.6	0.3	0.1	0	0	0.9
3	0.45	0.3	0.25	0	0	1.07
3	0.35	0.33	0.32	0	0	1.1
4	0.65	0.2	0.1	0.05	0	0.98
4	0.5	0.35	0.15	0.1	0	1.21
4	0.4	0.3	0.2	0.1	0	1.28
4	0.3	0.275	0.225	0.2	0	1.37
5	0.5	0.2	0.15	0.1	0.05	1.33
5	0.4	0.3	0.15	0.1	0.05	1.39
5	0.35	0.25	0.2	0.12	0.08	1.49
5	0.18	0.19	0.2	0.21	0.22	1.61

Supplementary Table 2: In-vitro mixtures. Proportion of DNA from each clone (A, B, C or D) in the 16 mixtures created. The QC column indicates whether the data passed quality control.

Mixture ID	A%	B%	C%	D%	QC	Number of clones	Shannon diversity
1	75	0	0	25	Pass	2	0.56
2	50	50	0	0	Pass	2	0.69
3	0	0	66.6	33.3	Pass	2	0.64
4	33.3	0	33.3	33.3	Pass	3	1.1
5	0	50	25	25	Pass	3	1.04
6	20	40	40	0	Pass	3	1.05
7	30	0	10	60	Pass	3	0.9
8	25	25	25	25	**Fail**	4	1.39
9	70	10	10	10	Pass	4	0.94
10	10	15	25	50	Pass	4	1.21
11	30	10	30	30	Pass	4	1.31
12	10	45	40	5	Pass	4	1.11
13	100	0	0	0	Pass	1	0
14	0	100	0	0	Pass	1	0
15	0	0	100	0	Pass	1	0
16	0	0	0	100	Pass	1	0

Supplementary Table 3: S score survival analysis. All p-values obtained using Cox proportional hazard models. Multivariate models comprised the percentage of genome altered (continuous variable) and the clinical stage of tumours (categorical variable). p stands for p value, CI stand for confidence interval for the hazard ratios. Significant associations are highlighted in bold.

	Overall survival					Relapse-free survival				
**Set**	**Samples**	**Univariate p**	**Univariate CI**	**Multivariate p**	**Multivariate CI**	**Samples**	**Univariate p**	**Univariate CI**	**Multivariate p**	**Multivariate CI**
**BLCA**	141	0.630	1.22 (0.55 - 2.70)	0.986	1.01 (0.44 - 2.33)	104	0.338	0.70 (0.33 - 1.46)	0.100	0.50 (0.22 - 1.14)
**BRCA**	865	0.223	1.32 (0.84 - 2.08)	0.329	1.30 (0.77 - 2.19)	594	0.277	1.29 (0.82 - 2.02)	0.492	1.20 (0.71 - 2.03)
**COAD**	396	**0.019**	**1.84 (1.11 - 3.06)**	0.503	1.21 (0.70 - 2.09)	342	**0.006**	**1.89 (1.21 - 2.97)**	0.309	1.28 (0.80 - 2.05)
**GBM**	452	**0.041**	**0.75 (0.56 - 0.99)**	NA	NA	292	0.212	0.82 (0.61 - 1.12)	NA	NA
**HNSC**	332	**0.003**	**2.00 (1.27 - 3.15)**	**0.046**	**1.65 (1.01 - 2.70)**	206	0.058	1.72 (0.98 - 3.01)	0.748	1.11 (0.58 - 2.13)
**KIRC**	467	0.063	1.46 (0.98 - 2.19)	0.937	0.98 (0.65 - 1.50)	129	0.174	1.40 (0.86 - 2.27)	0.757	1.08 (0.66 - 1.78)
**LAML**	144	0.388	1.28 (0.73 - 2.23)	NA	NA	NA	NA	NA	NA	NA
**LIHC**	149	0.753	0.91 (0.52 - 1.60)	0.734	0.90 (0.48 - 1.67)	136	0.579	1.14 (0.71 - 1.85)	0.898	1.03 (0.61 - 1.74)
**LUAD**	354	0.388	1.29 (0.73 - 2.27)	0.987	1.00 (0.56 - 1.77)	273	0.494	1.21 (0.70 - 2.07)	0.890	1.04 (0.60 - 1.79)
**LUSC**	247	0.227	0.66 (0.34 - 1.29)	0.345	0.72 (0.36 - 1.43)	147	0.710	0.86 (0.39 - 1.90)	0.586	0.80 (0.36 - 1.79)
**OV**	508	0.600	0.91 (0.63 - 1.31)	0.671	0.92 (0.64 - 1.33)	289	0.556	0.90 (0.63 - 1.28)	0.627	0.92 (0.64 - 1.30)
**PAAD**	63	0.772	0.85 (0.29 - 2.54)	0.534	0.69 (0.22 - 2.19)	62	0.629	1.26 (0.50 - 3.20)	0.919	0.95 (0.34 - 2.63)
**PRAD**	284	0.830	1.51 (0.03 - 66.09)	NA	NA	234	0.949	0.97 (0.37 - 2.53)	NA	NA
**SKCM**	241	**0.012**	**0.56 (0.35 - 0.88)**	0.148	0.70 (0.43 - 1.14)	235	**0.008**	**0.59 (0.40 - 0.87)**	0.091	0.70 (0.46 - 1.06)
**STAD**	138	**0.016**	**0.38 (0.17 - 0.84)**	0.108	0.50 (0.21 - 1.16)	136	**0.015**	**0.43 (0.22 - 0.85)**	0.089	0.53 (0.26 - 1.10)
**THCA**	297	0.124	0.31 (0.07 - 1.38)	0.386	0.52 (0.12 - 2.29)	237	0.232	0.55 (0.20 - 1.47)	0.656	0.80 (0.29 - 2.18)
**Pan-cancer**	5078	0.365	1.05 (0.94 - 1.17)	0.317	0.94 (0.84 - 1.06)	3416	**0.001**	**1.21 (1.08 - 1.35)**	0.333	1.06 (0.94 - 1.19)

Supplementary Table 4: R score survival analysis. All p-values obtained using Cox proportional hazard models. Multivariate models comprised the percentage of genome altered (continuous variable) and the clinical stage of tumours (categorical variable). p stands for p value, CI stand for confidence interval for the hazard ratios. Significant associations are highlighted in bold. *As the R score is inversely proportional to diversity, negative hazard ratios indicate an association between higher diversity and worse survival.

	Overall survival					Relapse-free survival				
**Set**	**Samples**	**Univariate p**	**Univariate CI***	**Multivariate p**	**Multivariate CI***	**Samples**	**Univariate p**	**Univariate CI***	**Multivariate p**	**Multivariate CI***
**BLCA**	141	0.977	1.00 (0.93 - 1.08)	0.845	1.01 (0.93 - 1.09)	104	0.187	1.06 (0.97 - 1.14)	0.066	1.08 (0.99 - 1.18)
**BRCA**	865	0.153	0.97 (0.92 - 1.01)	0.178	0.97 (0.92 - 1.02)	594	0.207	0.97 (0.93 - 1.02)	0.164	0.96 (0.91 - 1.02)
**COAD**	396	**0.045**	**0.95 (0.90 - 1.00)**	0.630	0.99 (0.93 - 1.05)	342	**0.049**	**0.95 (0.91 - 1.00)**	0.752	0.99 (0.94 - 1.04)
**GBM**	452	0.190	1.02 (0.99 - 1.04)	NA	NA	292	0.234	1.02 (0.99 - 1.05)	NA	NA
**HNSC**	332	**0.002**	**0.93 (0.89 - 0.98)**	**0.030**	**0.95 (0.90 - 0.99)**	206	**0.009**	**0.93 (0.87 - 0.98)**	0.155	0.95 (0.88 - 1.02)
**KIRC**	467	0.145	0.97 (0.92 - 1.01)	0.645	0.99 (0.94 - 1.04)	129	0.147	0.96 (0.90 - 1.02)	0.243	0.97 (0.91 - 1.02)
**LAML**	144	0.950	1.00 (0.95 - 1.06)	NA	NA	NA	NA	NA	NA	NA
**LIHC**	149	0.284	1.04 (0.97 - 1.11)	0.302	1.04 (0.97 - 1.11)	136	0.700	1.01 (0.95 - 1.07)	0.681	1.01 (0.95 - 1.08)
**LUAD**	354	0.266	0.97 (0.92 - 1.02)	0.622	0.99 (0.93 - 1.04)	273	0.507	0.98 (0.93 - 1.04)	0.802	0.99 (0.94 - 1.05)
**LUSC**	247	0.184	1.04 (0.98 - 1.11)	0.204	1.04 (0.98 - 1.11)	147	0.363	1.03 (0.96 - 1.11)	0.319	1.04 (0.96 - 1.12)
**OV**	508	0.742	0.99 (0.96 - 1.03)	0.584	0.99 (0.95 - 1.03)	289	0.751	1.01 (0.97 - 1.04)	0.919	1.00 (0.97 - 1.04)
**PAAD**	63	0.352	1.05 (0.94 - 1.18)	0.193	1.08 (0.96 - 1.22)	62	0.923	1.00 (0.91 - 1.10)	0.482	1.04 (0.94 - 1.15)
**PRAD**	284	0.891	0.97 (0.62 - 1.51)	NA	NA	234	0.859	1.01 (0.90 - 1.13)	NA	NA
**SKCM**	241	0.136	1.03 (0.99 - 1.08)	0.262	1.03 (0.98 - 1.08)	235	**0.042**	**1.04 (1.00 - 1.08)**	0.093	1.03 (0.99 - 1.08)
**STAD**	138	0.443	1.05 (0.93 - 1.18)	0.618	1.04 (0.90 - 1.19)	136	0.084	1.10 (0.99 - 1.22)	0.069	1.12 (0.99 - 1.26)
**THCA**	297	0.057	1.16 (1.00 - 1.34)	**0.035**	**1.17 (1.01 - 1.34)**	237	0.124	1.08 (0.98 - 1.18)	0.174	1.07 (0.97 - 1.17)
**Pan-cancer**	5078	0.674	1.00 (0.99 - 1.01)	0.337	1.01 (0.99 - 1.02)	3416	0.128	0.99 (0.98 - 1.00)	0.904	1.00 (0.99 - 1.01)

Supplementary Table 5: Percentage of genome altered survival analysis. All p-values obtained using Cox proportional hazard models. Multivariate models comprised the percentage of genome altered (continuous variable) and the clinical stage of tumours (categorical variable). p stands for p value, CI stand for confidence interval for the hazard ratios. Significant associations are highlighted in bold. 8 samples for which copy number data was available but with no LogR and BAF data were included in this table (2 GBM, 4 HNSC, 2 OV).

	Overall survival					Relapse-free survival				
**Set**	**Samples**	**Univariate p**	**Univariate CI**	**Multivariate p**	**Multivariate CI**	**Samples**	**Univariate p**	**Univariate CI**	**Multivariate p**	**Multivariate CI**
**BLCA**	141	0.556	1.51 (0.39 - 5.88)	0.788	1.21 (0.30 - 4.90)	104	0.902	0.92 (0.25 - 3.39)	0.497	0.61 (0.15 - 2.53)
**BRCA**	865	**0.009**	**3.51 (1.37 - 8.96)**	**0.006**	**4.30 (1.52 - 12.18)**	594	0.210	1.79 (0.72 - 4.47)	0.222	1.89 (0.68 - 5.28)
**COAD**	396	0.674	1.23 (0.47 - 3.18)	0.118	0.43 (0.15 - 1.24)	342	0.202	1.76 (0.74 - 4.17)	0.384	0.65 (0.25 - 1.70)
**GBM**	454	0.322	0.69 (0.33 - 1.44)	NA	NA	293	0.916	0.95 (0.37 - 2.45)	NA	NA
**HNSC**	336	**0.003**	**3.42 (1.52 - 7.71)**	0.064	2.27 (0.95 - 5.43)	207	0.097	2.40 (0.85 - 6.76)	0.508	1.50 (0.45 - 5.04)
**KIRC**	467	**0.012**	**2.53 (1.22 - 5.21)**	0.328	1.46 (0.68 - 3.11)	129	0.153	1.84 (0.80 - 4.24)	0.929	0.96 (0.37 - 2.45)
**LAML**	144	**0.015**	**29.91 (1.96 - 456.4)**	NA	NA	NA	NA	NA	NA	NA
**LIHC**	149	0.330	1.69 (0.59 - 4.82)	0.662	1.31 (0.40 - 4.31)	136	**0.008**	**3.43 (1.38 - 8.51)**	0.071	2.54 (0.92 - 7.00)
**LUAD**	354	0.298	1.65 (0.64 - 4.23)	0.833	0.91 (0.36 - 2.29)	273	0.273	1.68 (0.67 - 4.23)	0.664	1.23 (0.48 - 3.15)
**LUSC**	247	0.542	0.65 (0.17 - 2.57)	0.417	0.56 (0.14 - 2.26)	147	0.964	0.97 (0.21 - 4.46)	0.811	0.82 (0.17 - 4.00)
**OV**	510	0.315	1.50 (0.68 - 3.28)	0.240	1.63 (0.72 - 3.71)	290	0.241	1.63 (0.72 - 3.69)	0.133	1.90 (0.82 - 4.38)
**PAAD**	63	0.200	3.02 (0.56 - 16.43)	0.330	2.59 (0.38 - 17.65)	62	0.061	3.87 (0.94 - 15.90)	0.197	2.94 (0.57 - 15.11)
**PRAD**	284	0.546	9.18 (0.01 - 12371)	NA	NA	234	**0.008**	**13.06 (1.95 - 87.56)**	NA	NA
**SKCM**	241	0.083	0.46 (0.19 - 1.10)	0.373	0.63 (0.22 - 1.76)	235	**0.028**	**0.43 (0.21 - 0.91)**	0.175	0.55 (0.23 - 1.31)
**STAD**	138	0.349	0.57 (0.18 - 1.84)	0.430	0.62 (0.19 - 2.04)	136	0.483	0.69 (0.25 - 1.93)	0.721	0.83 (0.29 - 2.35)
**THCA**	297	0.809	1.80 (0.02 - 209.0)	0.501	7.26 (0.02 - 2354.1)	237	0.932	1.16 (0.04 - 35.90)	0.531	3.33 (0.08 - 144.2)
**Pan-cancer**	5086	**0.0002**	**1.43 (1.18 - 1.72)**	0.427	1.09 (0.88 - 1.34)	3416	**9.0E-12**	**1.98 (1.63 - 2.41)**	**0.032**	**1.26 (1.02 - 1.56)**

Supplementary Figures

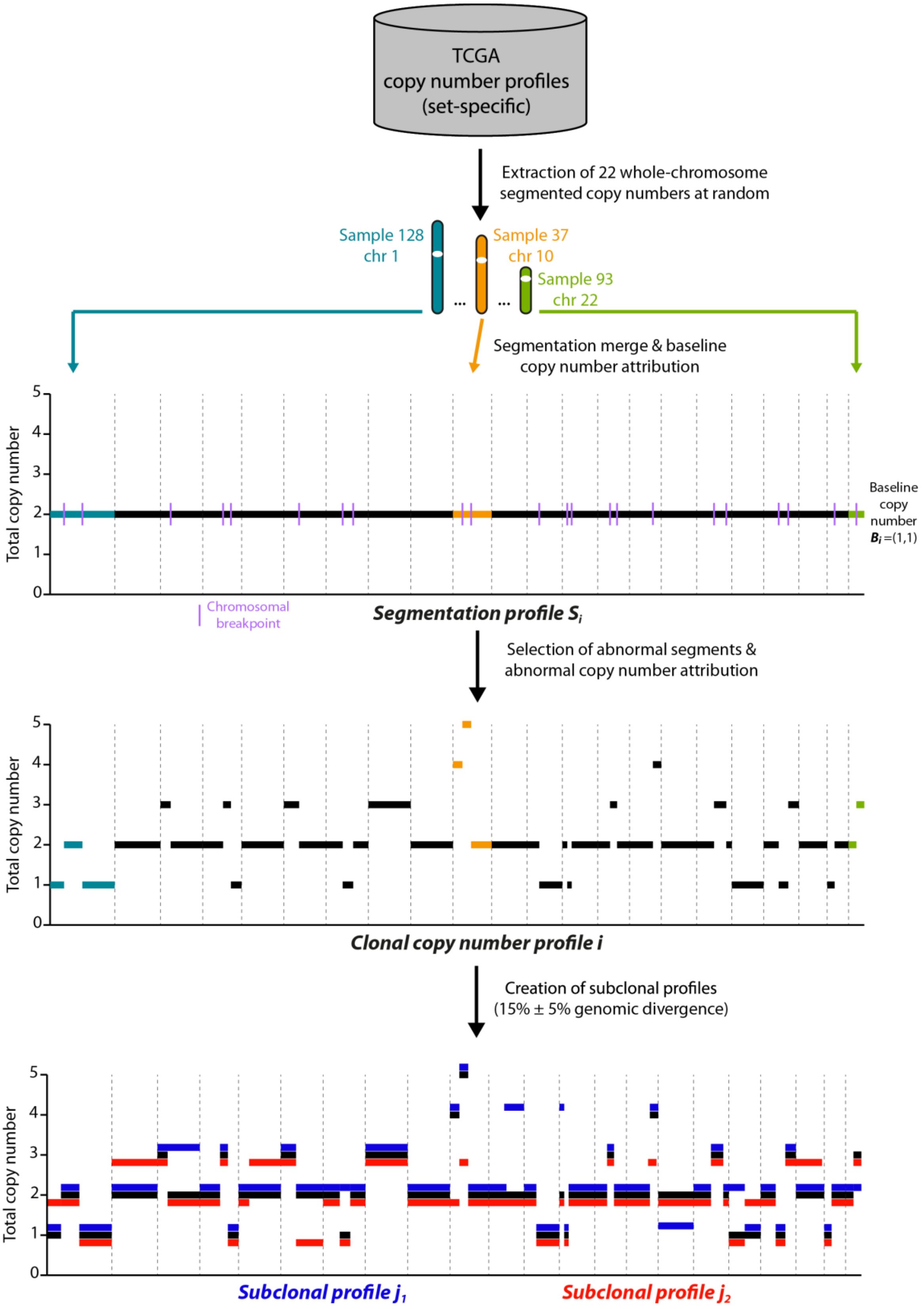

Supplementary Figure 1. Generation of clonal and subclonal synthetic profiles. For each of the 22 autosomes, a list of segments and their breakpoints S_i is selected at random from any TCGA sample of the same cancer type. They are merged in respective order and their copy number is set to a baseline B_i (here 1 copy of each allele), drawn from the cancer type specific TCGA baseline copy number distribution. A subset of segments is selected at random up to a randomly drawn percentage of abnormal genome. Their copy numbers are individually set to an abnormal number to constitute a clonal profile (in black). For each clonal profile *i*, 4 subclonal profiles $j_{1\text{-}4}$ are created by adding some divergent copy number states to the initial clonal profile, so that the genomes differ by 15% ± 5%. For clarity, only 2 subclonal profiles are shown (in red and blue). Vertical dotted lines in copy number profiles indicate chromosome boundaries.

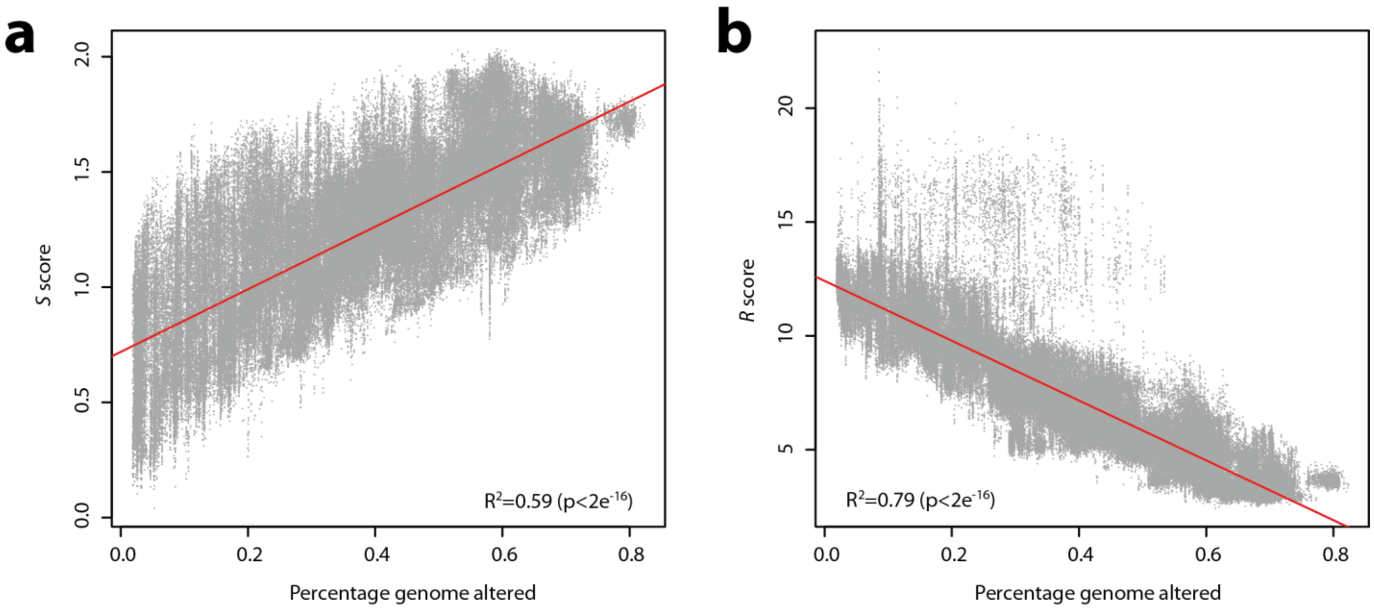

Supplementary Figure 2. Correlation of the S and R scores with the percentage of genome altered in in-silico mixtures. a) S score. b) R score. Grey dots indicate individual simulated profiles. Red lines indicate the linear fit between the scores and the percentage of genomes altered.

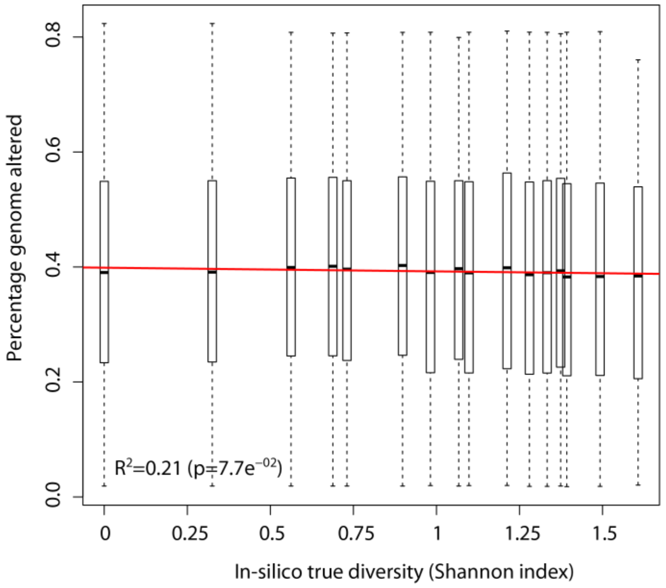

Supplementary Figure 3. Relationship between percentage of genome altered and true diversity in in-silico mixtures. The red line indicates the linear fit between the median *S* scores and the expected diversity. R^2=0.00, p=0.70. The percentage of genome altered is independent of the mixture cellularity.

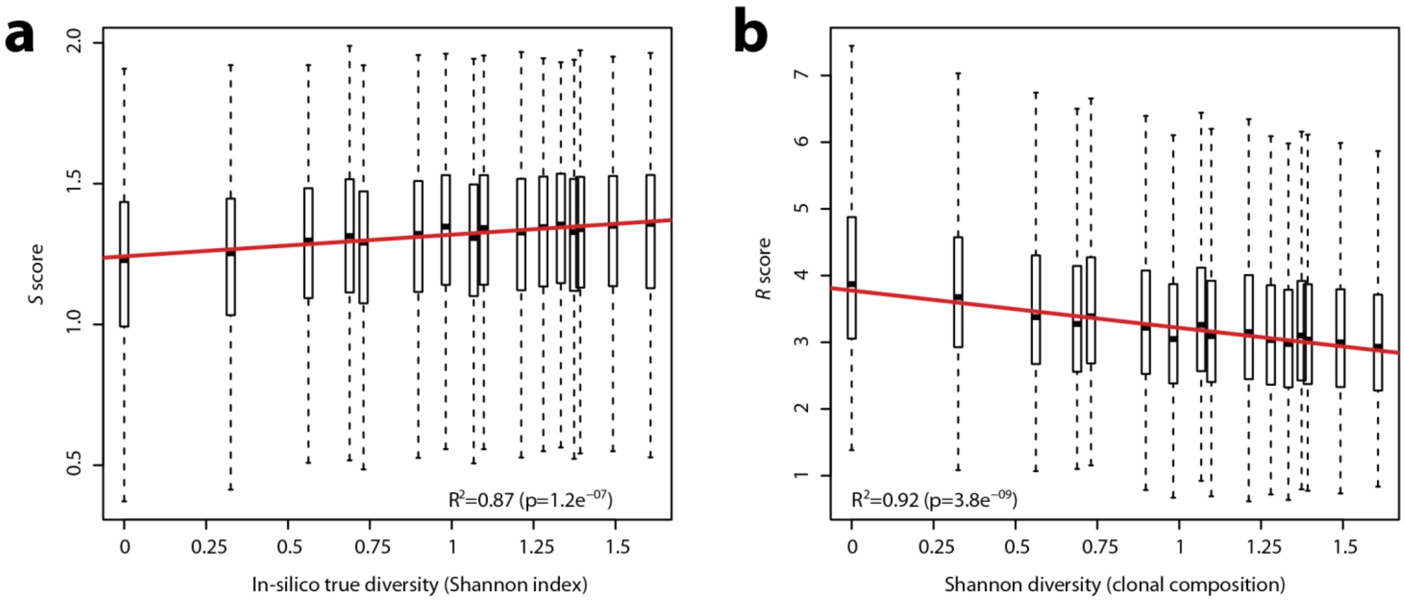

Supplementary Figure 4. Relationship between *S* score, *R* score and true diversity in low-cellularity in-silico mixtures. a) *S* score. b) *R* score. The red line indicates the linear fit between the median scores and the expected diversity.

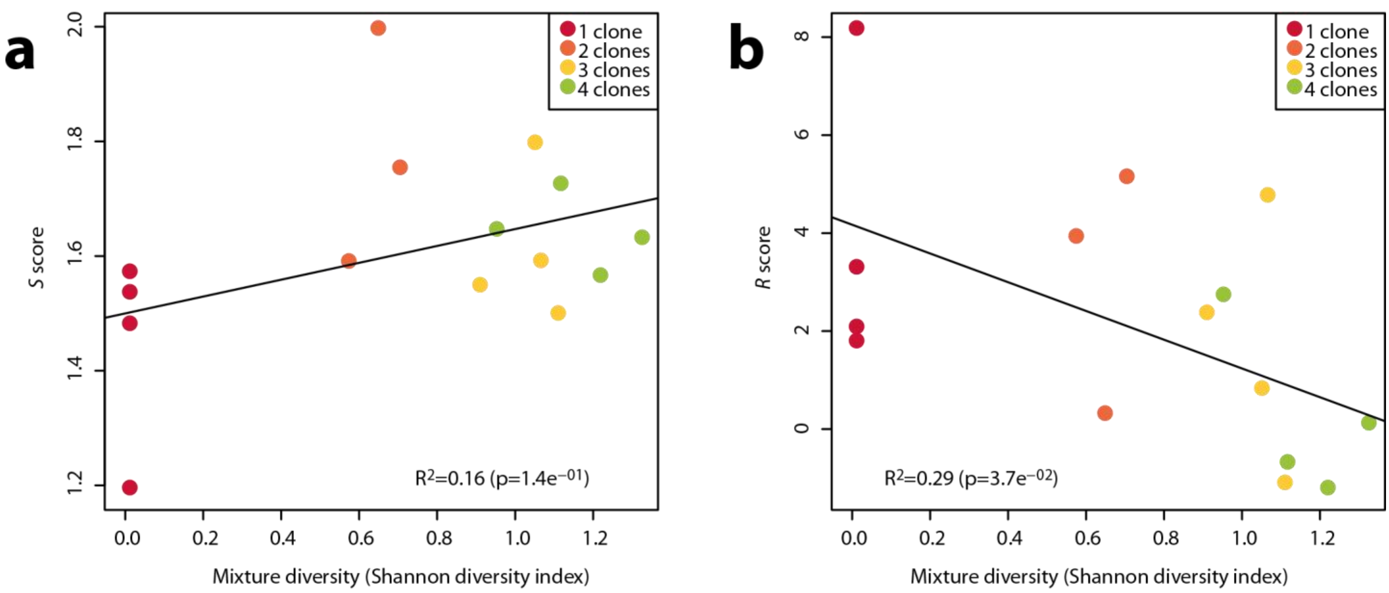

Supplementary Figure 5. Relationship between *S* and *R* scores and true diversity in in-vitro mixtures. The black line indicates the linear fit between the scores and the expected diversity.

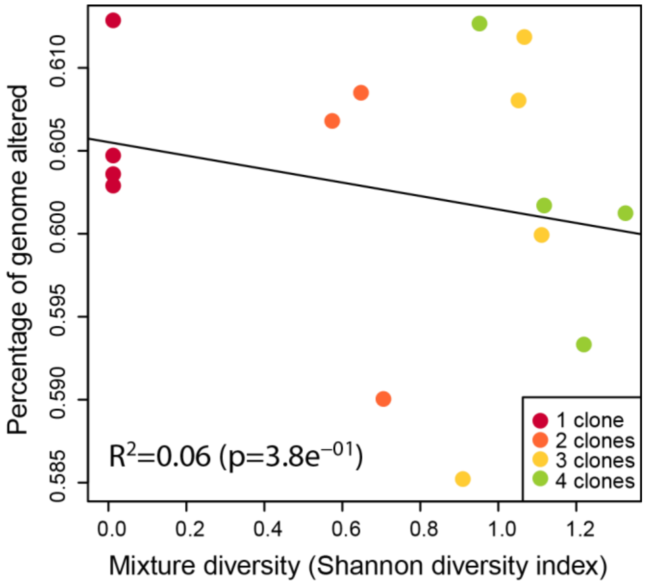

Supplementary Figure 6. Relationship between percentage of genome altered and true diversity in in-vitro mixtures. The black line indicates the linear fit between the scores and the expected diversity.

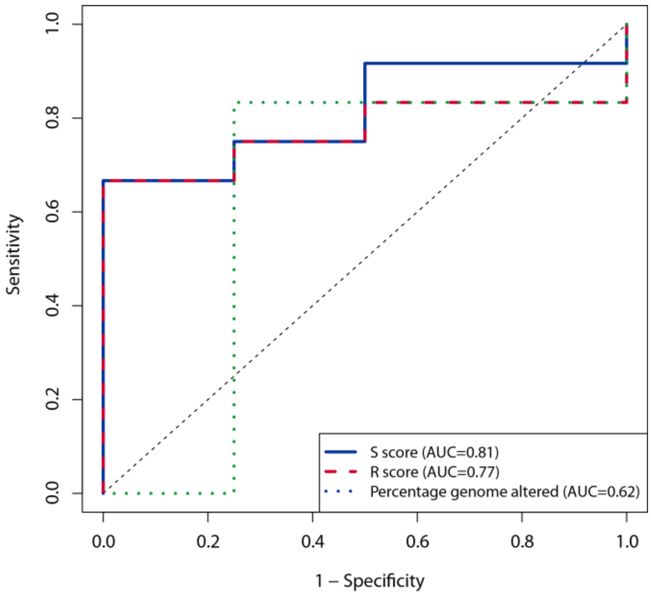

Supplementary Figure 7. In-vitro Area Under the Curve. Dotted black lines indicates expectations specificity and sensitivity values for random prediction. Coloured lines correspond to the Receiver Operator Curves obtained for the *S* score (blue), *R* score (red) and percentage of genome altered (green).

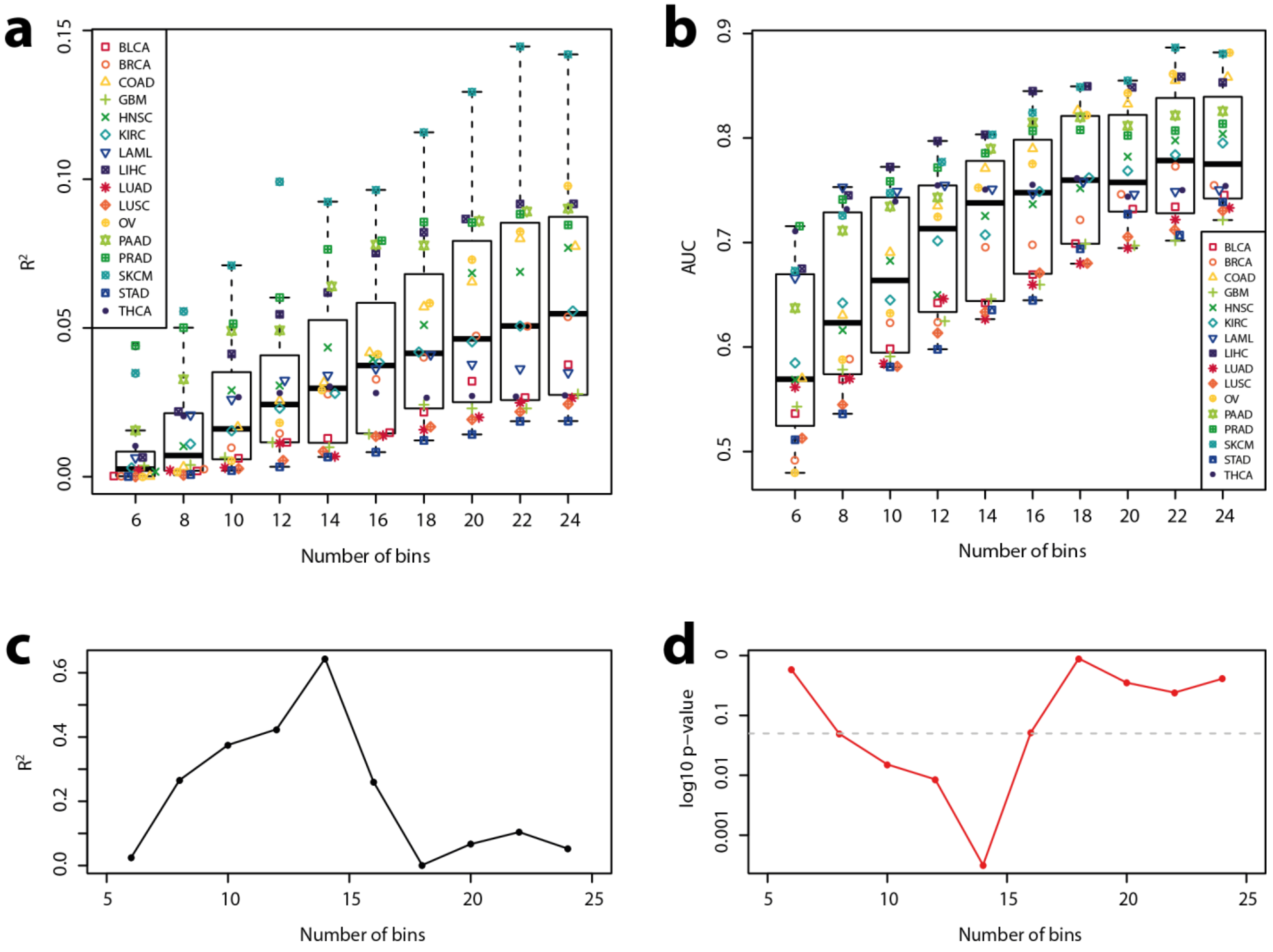

Supplementary Figure 8: *S* score calibration using in-silico and in-vitro data. a) Distributions of goodness of fit (R^2) between *S* scores calculated using different numbers of bins and true in-silico diversity, per set. B) Distributions of Area Under the Curve (AUC) to determine the power to discriminate between monoclonal and polyclonal samples in in-silico data, based on different numbers of bins on a per-set basis. c) Distribution of the goodness of fit (R^2) between the *S* score calculated using different numbers of bins and true in-vitro diversity. d) Distribution of p-values for the correlation test between the *S* score calculated using different numbers of bins and true in-vitro diversity.

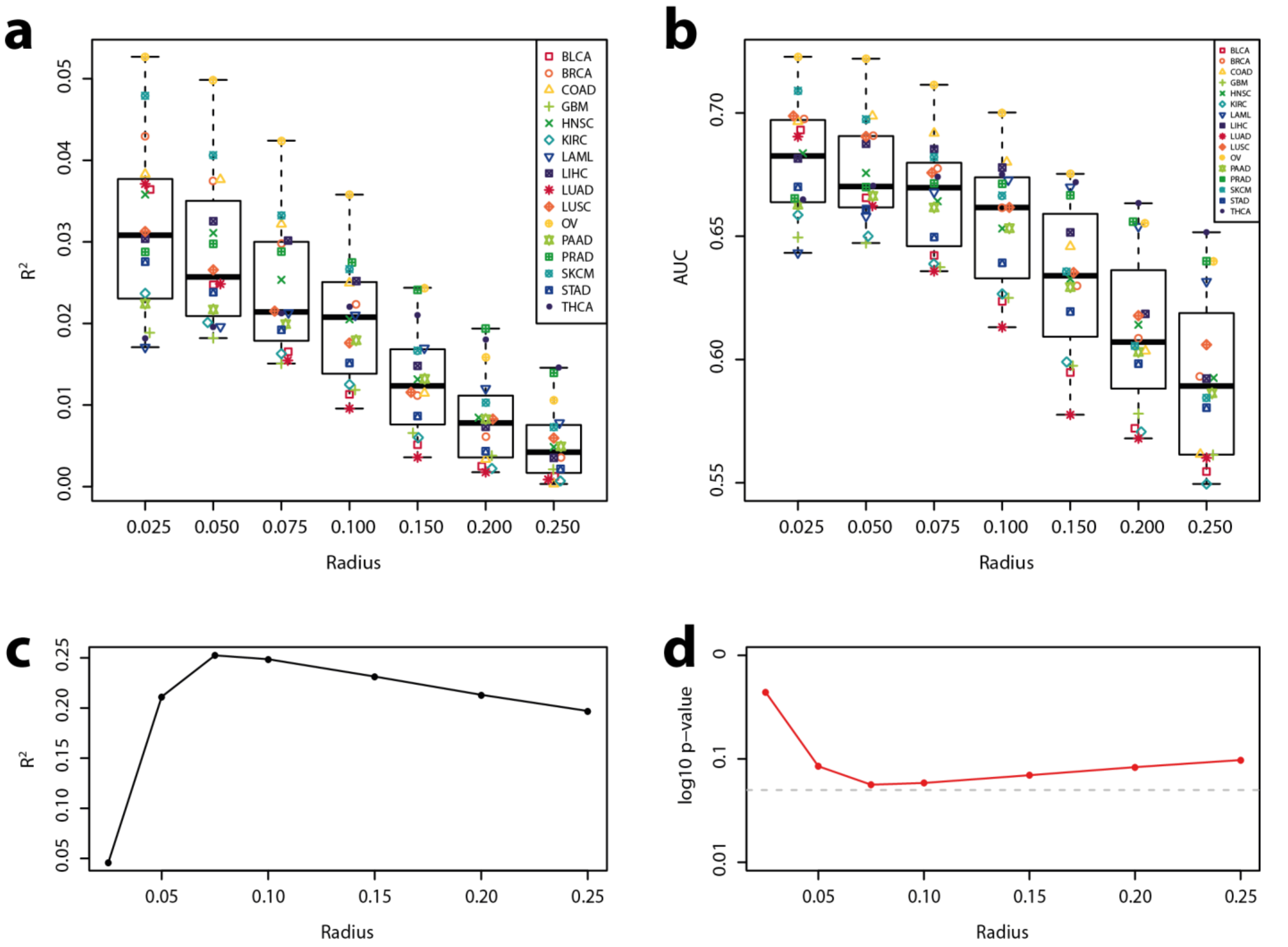

Supplementary Figure 9: *R* score calibration using in-silico and in-vitro data. a) Distributions of goodness of fit (R^2) between *R* scores calculated using different numbers of bins and true in-silico diversity, per set. B) Distributions of Area Under the Curve (AUC) to determine the power to discriminate between monoclonal and polyclonal samples in in-silico data, based on different numbers of bins on a per-set basis. c) Distribution of the goodness of fit (R^2) between the *R* score calculated using different numbers of bins and true in-vitro diversity. d) Distribution of p-values for the correlation test between the *R* score calculated using different numbers of bins and true in-vitro diversity.

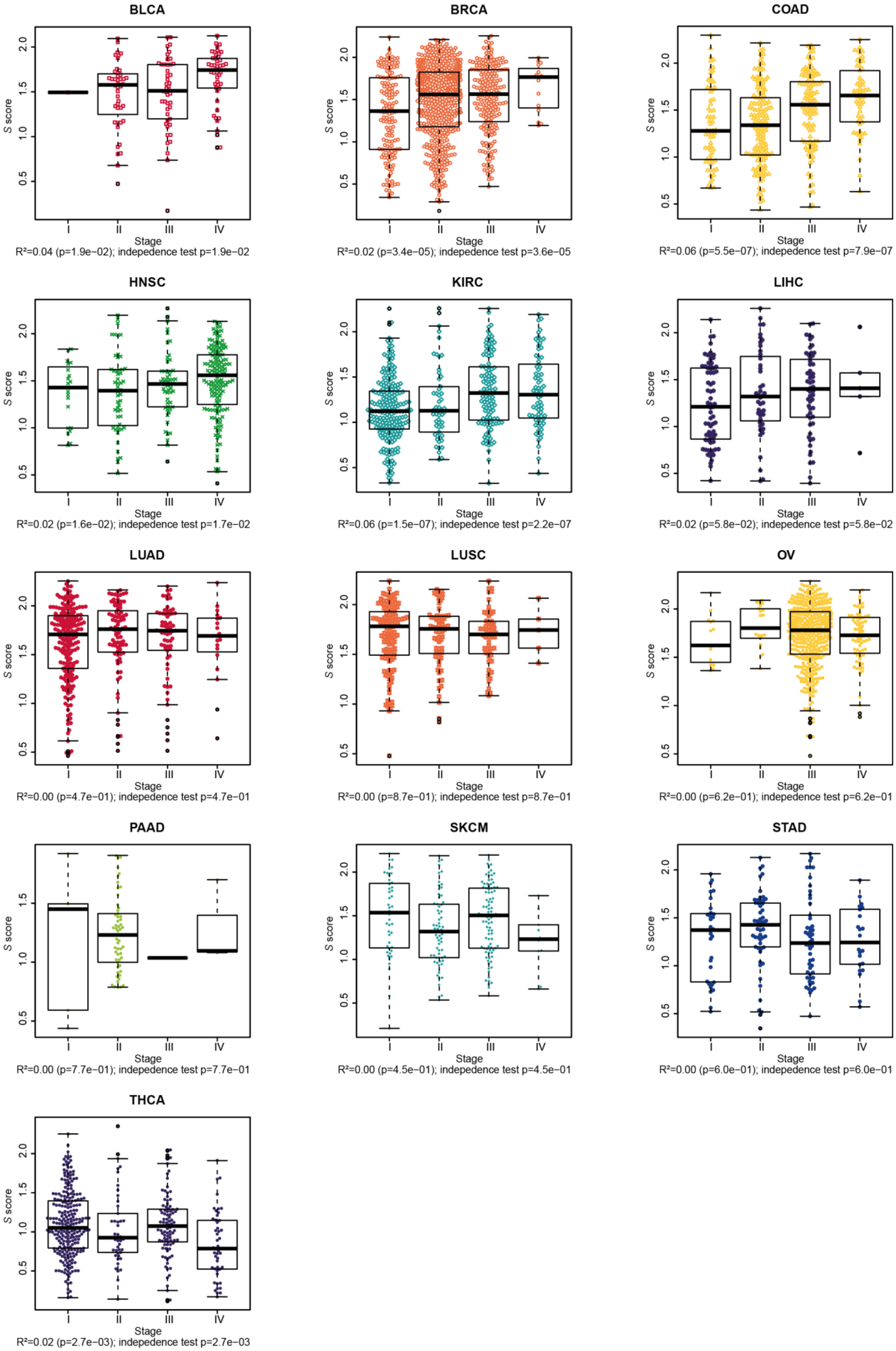

Supplementary Figure 10: Distributions of the *S* score per cancer stage in 13 cancer types. Coloured dots represent individual samples.

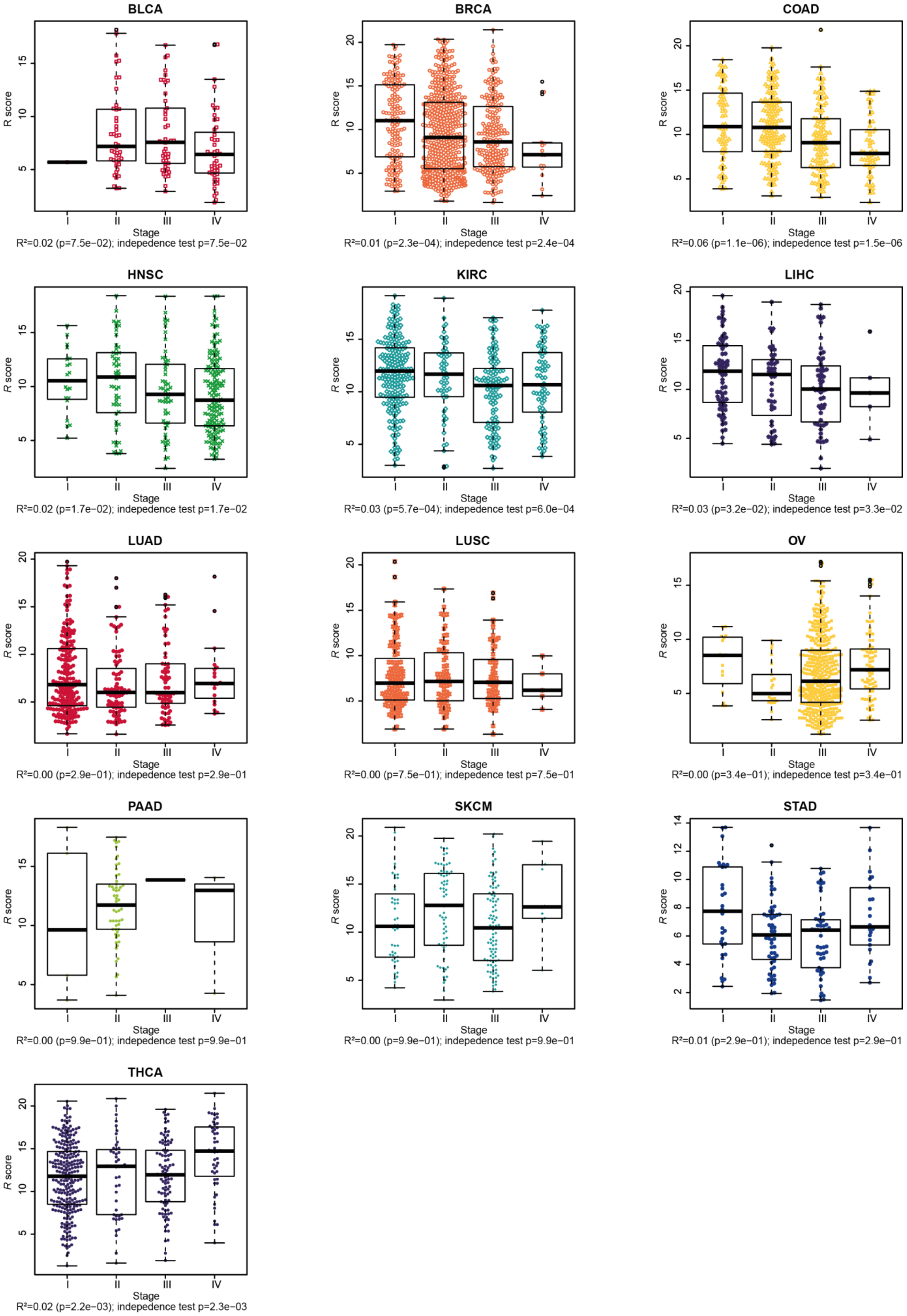

Supplementary Figure 11: Distributions of the *R* score per cancer stage in 13 cancer types. Coloured dots represent individual samples.

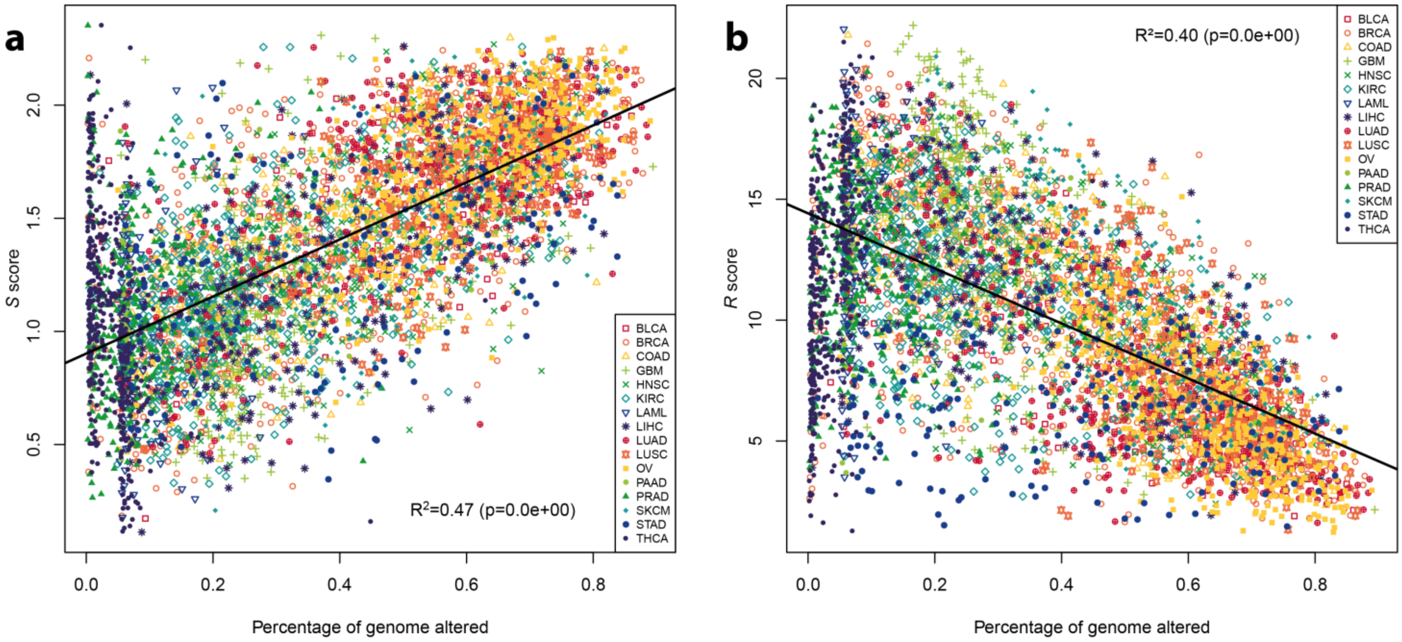

Supplementary Figure 12: Correlation between *S* score, *R* score and percentage of genome altered. a) *S* score. b) *R* score. Black line indicates the linear fit between both measures. Different styles and colours for dots indicate different cancer types.

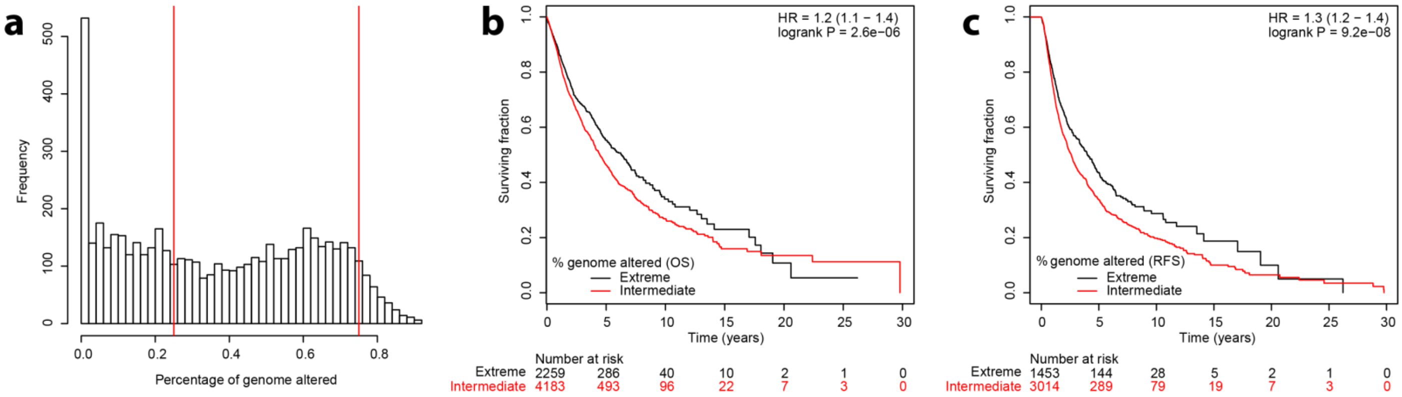

Supplementary Figure 13. Intermediate and extreme percentage of genome altered. a) Histogram of the percentage of genome altered in 5,086 TCGA samples. Vertical red lines indicate the boundaries of the intermediate (≥25% and ≤75%) and extreme (<25% and >75%) categories. b-c) Overall (b) and relapse-free (c) survival analysis using Kaplan-Meier curves.

Algorithm implementation

```
snpDivCols <- c("SampleID", "Chr", "Start", "End", "nProbes", "mBAF", "logR")
methodV <- c("ripley", "shannon", "simpson")
library(vegan)
library(spatstat)
library(parallel)

## Main function. Options discussed below:
## data: Input data object (matrix of segments, described below).
## minprobes: Minimum number of probes for a segment to be included in the calculation (default 100).
## minkb: Minimum segment size (in kb) for a segment to be included in the calculation (default 1000, i.e. 1Mb).
## nbins: Number of bins for the calculation of logR-based shannon diversity (S score, default 10).
## method: Which measure is to be calculated, either "shannon" (default) for the Shannon diversity based S score,
##         "simpson" for a Simpson diversity alternative or "ripley" for the Ripley's L-based L score.
## norm: Should the data be normalised from 0 to 1 (default TRUE).
## delog: Should the logRs be converted to floating point ratio estimates (2^logR, default FALSE).
## ncores: Number of cores to be used (default 1). If more than 1, mclapply from the parallel package will be used.
## fixedmin: Minimum value used for capping logRs. Not set by default. If set, will be considered as minimum possible
##           value for bin creation.
## fixedmax: Maximum value used for capping logRs. Not set by default. If set, will be considered as maximum possible
##           value for bin creation.
## maxrad: Maximum radius for the Ripley L calculation (R score, default 0.05).
## radinc: Radius increment for the Ripley L calculation (R score, default 0.001).
##
## Format of input 'data' object
## 7-Column matrix: SampleID, Chr, Start, End, nProbes, mBAF, logR
## Each row corresponds to a genomic segment
## SampleID: A unique identifier for each sample
## Chr: The chromosome on which the segment is located
## Start: Location of the first base pair of the segment on the chromosome
## End: Location of the last base pair of the segment on the chromosome
## nProbes: Number of probes covered by the segment
## mBAF: Mean mirrored B Allele Frequency value of all probes in the segment
## logR: Mean logR value of all probes in the segment
snpDiv <- function(data, minprobes=100, minkb=1000, nbins=10, method="shannon", norm=TRUE, delog=FALSE, ncores=1,
                   fixedmin=NA, fixedmax=NA, maxrad=0.05, radinc=0.001) {
  ## Argument check
  if (length(grep(FALSE, snpDivCols %in% colnames(data))) != 0) {
    print(paste("Error, data must contain the following columns:", paste(snpDivCols, collapse=" ")))
    return(NULL)
  }
  if (as.numeric(minprobes) < 1 | is.na(as.numeric(minprobes))) {
    print("Error: minprobes argument must be numeric and > 0")
    return(NULL)
  }
  if (as.numeric(minkb) < 0.001 | is.na(as.numeric(minkb))) {
    print("Error: minkb argument must be numeric and >= 0.001")
    return(NULL)
  }
  if (method %in% c("shannon", "simpson")) {
    if (as.numeric(nbins) <= 2 | is.na(as.numeric(nbins))) {
      print("Error: nbins argument must be numeric and >= 2")
      return(NULL)
    }
  }
  if (!method %in% methodV) {
    print(paste("Error, possible values for method argument are:", paste(methodV, collapse=" ")))
    return(NULL)
  }

  ## Create data frame
  segments=data.frame(SampleID=as.character(data[,"SampleID"]),
                      Chr=as.numeric(data[,"Chr"]), Start=as.numeric(data[,"Start"]), End=as.numeric(data[,"End"]),
                      nProbes=as.numeric(data[,"nProbes"]), mBAF=as.numeric(data[,"mBAF"]),
                      logR=as.numeric(data[,"logR"]))
  segments <- segments[which(segments$nProbes >= minprobes &
                             ((segments$End - segments$Start) >= (minkb * 1000))),]

  ## Sanity checks
  ## mean BAFs should be mirrored (>0.5). Do it if it's not the case.
  if (method == "ripley") {
    tmpidx <- which(segments$mBAF < 0.5)
    if (length(tmpidx) > 0) {
      print("Warning: some segments have mBAF < 0.5 - shouldn't be the case. These will be mirrored.")
      segments$mBAF[tmpidx] <- 0.5 + abs(segments$mBAF[tmpidx] - 0.5)
    }
  }

  ## Capping logRs if needed
  if (!is.na(fixedmin)) {
```

```
    segments$logR <- unlist(lapply(segments$logR, function(x){max(x,fixedmin)}))
  }
  if (!is.na(fixedmax)) {
    segments$logR <- unlist(lapply(segments$logR, function(x){min(x,fixedmax)}))
  }

  ## Setting maxrad to 0.05 if undefined
  if (is.na(as.numeric(maxrad))) {
    maxrad=0.05
  }
  if (is.na(as.numeric(radinc))) {
    maxrad=0.001
  }

  ## Delog logRs if asked
  if (delog == T) {
    segments$logR <- 2 ^ segments$logR
  }

  sids <- unique(segments$SampleID)

  if (ncores == 1) {
    res <- unlist(lapply(sids, calcSnpDiv, segments=segments, nbins=nbins, method=method, norm=norm,
                         maxrad=maxrad, radinc=radinc, fixedmin=fixedmin, fixedmax=fixedmax))
  } else {
    res <- unlist(mclapply(sids, calcSnpDiv, segments=segments, nbins=nbins, method=method, norm=norm,
                           maxrad=maxrad, radinc=radinc, fixedmin=fixedmin, fixedmax=fixedmax, mc.cores=ncores))
  }
  names(res) <- sids
  return(res)
}

## Calculate the R score
calcRipleysL <- function(segmentList, norm, maxrad, radinc) {
  if (norm == T) {
    ## convert segmentList to a "planer point pattern" for spapstat
    ## normalise BAFs and logRs
    segmentList[,"mBAF"] <- segmentList[,"mBAF"] - min(segmentList[,"mBAF"], na.rm=T)
    segmentList[,"mBAF"] <- segmentList[,"mBAF"] / max(segmentList[,"mBAF"], na.rm=T)
    segmentList[,"logR"] <- segmentList[,"logR"] - min(segmentList[,"logR"], na.rm=T)
    segmentList[,"logR"] <- segmentList[,"logR"] / max(segmentList[,"logR"], na.rm=T)
    ## convert segmentList to a "planer point pattern" for spapstat
    pp <- as.ppp(segmentList, c(0,1,0,1))
    ## calc Ripley's K
    ripleyL <- Lest(pp,correction="isotropic", r=seq(0, maxrad, radinc))
  } else {
    ## convert segmentList to a "planer point pattern" for spapstat. Limit to 0:1 for BAF, -3:3 for logR.
    pp <- as.ppp(segmentList, c(0,1,-3,3))
    ## calc Ripley's K
    ripleyL <- Lest(pp,correction="isotropic");
    ## calc difference from theoretical (Poisson) random process
  }
  return(ripleyL)
}

## Calculate number of values in given bin
ninbin <- function(bin, data, binmin, binmax, binsize) {
  length(which(data >= seq(binmin, binmax, binsize)[bin] &
               data < seq(binmin, binmax, binsize)[bin+1]))
}

## Subfunction, calculate the score per sample.
calcSnpDiv <- function(sampleid, segments, nbins, method, norm, maxrad, radinc, fixedmin, fixedmax) {
  ##print(sampleid)
  sidsegs <- segments[which(segments$SampleID == sampleid),]

  ## Ripley's L
  if (method == "ripley") {
    sidsegs <- sidsegs[which(!is.na(sidsegs$mBAF) & !is.na(sidsegs$logR)),,drop=F]
    if (nrow(sidsegs) == 0) {
      return(NA)
    }
    tmp <- calcRipleysL(sidsegs[,c("mBAF", "logR")], norm=norm, maxrad, radinc)
    return(sum(tmp$iso-tmp$theo))
  }

  ## Shannon/simpson diversity indexes
  if (method %in% c("shannon", "simpson")) {
    sidsegs <- sidsegs[which(!is.na(sidsegs$mBAF) & !is.na(sidsegs$logR)),,drop=F]
    if (nrow(sidsegs) == 0) {
      return(NA)
    }
```

```
    stat = sidsegs$logR
    binmax=ifelse(is.na(fixedmax), max(stat, na.rm=T), fixedmax)
    binmin=ifelse(is.na(fixedmin), min(stat, na.rm=T), fixedmin)
    binsize <- (binmax-binmin)/nbins
    bins <- unlist(lapply(1:nbins, ninbin, data=stat, binmin=binmin, binmax=binmax, binsize=binsize))
    bins[nbins] = bins[nbins] + length(which(stat==binmax))
    ## Return diversity method (vegan library)
    return(diversity(bins, index=method))
  }
  return(NA)
}
```
